# IBR1, a novel endogenous IFIH1‐binding dsRNA, governs IFIH1 activation and M1 macrophage polarisation in ARDS

**DOI:** 10.1002/ctm2.70027

**Published:** 2024-09-23

**Authors:** Shi Zhang, Wei Huang, Xueling Wu, Hanbing Chen, Lu Wang, Jie Chao, Jianfeng Xie, Haibo Qiu

**Affiliations:** ^1^ Jiangsu Provincial Key Laboratory of Critical Care Medicine Department of Critical Care Medicine Zhongda Hospital School of Medicine Southeast University Nanjing China; ^2^ Department of Respiratory and Critical Care Medicine Central Hospital Affiliated to Shandong First Medical University Jinan China; ^3^ Department of Respiratory and Critical Care Medicine Renji Hospital Shanghai Jiao Tong University School of Medicine Shanghai China

**Keywords:** ARDS, dsRNA, IFIH1, novel transcript

## Abstract

**Background:**

Uncontrolled inflammation caused by macrophages and monocytes plays a crucial role in worsening acute respiratory distress syndrome (ARDS). Previous studies have highlighted the importance of IFIH1 in regulating macrophage polarisation in ARDS triggered by pneumonia. However, the mechanisms by which IFIH1 is activated in ARDS remain unclear.

**Methods:**

In this study, we utilised multiomics sequencing and molecular interaction experiments to explore the molecular mechanisms underlying IFIH1 activation in ARDS. Through the use of conditional gene knockout mice and primary cells, we demonstrated the significant role of these mechanisms in the development of ARDS. Additionally, we validated the associations between these mechanisms and ARDS by quantitative PCR analysis of CD14^+^ cells obtained from the peripheral blood of 140 ARDS patients.

**Results:**

Our investigation revealed that lipopolysaccharide, a critical component derived from Gram‐negative bacteria, activated IFIH1 by upregulating a novel transcript known as IFIH1‐binding RNA1 (IBR1) in monocytes and macrophages. Specifically, as an endogenous double‐stranded RNA, IBR1 bind to the helicase domain of IFIH1 because of its unique double‐stranded structure. Deletion of IBR1 significantly reduced the activation of IFIH1, M1 polarisation of macrophages, and inflammatory lung injury in ARDS. Moreover, IBR1 directly induced M1 polarisation of macrophages and ARDS, whereas deletion of IFIH1 inhibited IBR1‐induced macrophage M1 polarisation and inflammatory lung injury. Importantly, we observed a notable increase in IBR1 expression in ARDS patients with pneumonia caused by Gram‐negative bacteria. Furthermore, we demonstrated that the delivery of IFIH1 mutants through exosomes effectively counteracted IBR1, thereby reducing pulmonary inflammation and alleviating lung injury.

**Conclusions:**

This study revealed a novel mechanism involving IBR1, an endogenous double‐stranded RNA (dsRNA) that binds to IFIH1, shedding light on the complex process of macrophage polarisation in ARDS. The administration of IFIH1 variants has the potential to eliminate pulmonary dsRNA and alleviate inflammatory lung injury in ARDS.

**Highlights:**

1. In monocytes and macrophages, the endogenous double‐stranded RNA, IFIH1‐binding RNA 1 (IBR1), binds to the helicase domain of IFIH1 because of its unique double‐stranded structure.
2. IBR1 plays a significant role in macrophage polarisation and the development of acute respiratory distress syndrome (ARDS) induced by Gram‐negative bacteria or lipopolysaccharide (LPS).
3. Administration of IFIH1 variants has potential for eliminating pulmonary IBR1 and reducing inflammatory lung injury in ARDS patients.

## Graphical abstract

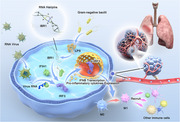

## 1 INTRODUCTION

Acute respiratory distress syndrome (ARDS) is a severe type of respiratory failure that accounts for 10% of admissions to intensive care units. Despite advancements in both basic science and clinical research, therapeutic options for ARDS remain limited, with severe cases continuing to have a high mortality rate.^1^, ^2^ Consequently, there is an urgent imperative to enhance our comprehension of disease pathogenesis and to innovate novel treatment modalities.

Uncontrolled inflammatory responses within the pulmonary milieu, orchestrated predominantly by macrophages and monocytes, assume a central role in perpetuating lung injury. Alveolar macrophages and recruited monocytes have can be activated into proinflammatory populations, known as M1 macrophage polarisation, which exacerbates pulmonary inflammation and lead to prolonged lung injury.^3^, ^4^, ^5^, ^6^, ^7^ Thus, it is crucial to investigate macrophage polarisation towards the proinflammatory phenotype and modulate the activity during the nascent stages of ARDS.

Our previous research clarified the critical role of IFIH1 in orchestrating macrophage M1 polarisation during pneumonia‐induced ARDS.^8^, ^9^, ^10^ IFIH1, a cytosolic receptor, is primarily responsible for detecting viral double‐stranded RNA (dsRNA), which triggers signalling cascades involved in both inflammatory and antiviral responses.^11^, ^12^, ^13^ Studies have shown that upon binding to dsRNA, the helicase domain of the IFIH1 protein catalyses ATP hydrolysis, facilitating the interaction between the CARD domain of IFIH1 and the MAVS protein. The resulting IFIH1‒MAVS complex then promotes the phosphorylation and nuclear translocation of the IRF3/IRF7 transcription factors, which increase the transcription of interferons and inflammatory cytokines.

Common methods used to verify IFIH1 activation include ATP hydrolysis assays and Western blot analysis for IRF3/IRF7 phosphorylation. In vitro, ATP hydrolysis experiments are primarily used to assess IFIH1 activation, as successful ATP hydrolysis indicates IFIH1 activation. Conversely, in vivo studies—both cellular and animal experiments—often focus on measuring the phosphorylation levels of IRF3/IRF7 or their nuclear expression, where significant phosphorylation or high nuclear levels suggest IFIH1 activation.

However, our findings indicated that in patients with ARDS induced by bacterial pneumonia (in the absence of exogenous dsRNA stimulation), there was a persistent IFIH1‐mediated excessive inflammatory response in CD14^+^ cells. The mechanisms by which IFIH1 is activated by bacteria remain poorly understood. Investigating these molecular mechanisms could provide valuable insights for developing immunotherapeutic strategies for ARDS.

Our investigation revealed that the pivotal pathogenic component lipopolysaccharide (LPS) derived from Gram‐negative bacilli activates IFIH1 by upregulating a novel transcript, IFIH1‐binding RNA1 (IBR1), within monocytes and macrophages. IBR1 interacted with the IFIH1 protein, forming a complex, and functioned as an endogenous dsRNA that specifically bind to the helicase domain of IFIH1 because of its localised double‐stranded configuration. This interaction activated IFIH1, leading to the induction of macrophage M1 polarisation in the context of inflammatory lung injury. Notably, elevated IBR1 expression was observed in ARDS patients with Gram‐negative bacilli‐induced pneumonia. Exosome‐mediated delivery of IFIH1 mutants effectively neutralised IBR1, thereby attenuating lung inflammation. This study not only elucidates molecular interactions but also delineates potential therapeutic interventions for ARDS.

## 2 MATERIALS AND METHODS

### 2.1 ARDS patients

The collection of human samples was approved by the Institutional Ethics Committee of Zhongda Hospital and the Central Hospital Affiliated with Shandong First Medical University.

Patients with pneumonia‐induced ARDS were selected from two different locations a group from the ARDS biobank at the Affiliated Zhongda Hospital of Southeast University, and the rest from ARDS patients at the Center Hospital.

#### 2.1.1 Inclusion criteria

Age greater than or equal to 18 years old; diagnosis of ARDS in accordance with the 2012 Berlin definition; onset of ARDS within 48 h; ARDS caused by pneumonia; presence of significant inflammatory infiltrative lesions in the lung imaging of the case group (including patchy shadows, large patchy ground‐glass opacities, lobar or segmental consolidation).

#### 2.1.2 Exclusion criteria

The following conditions are considered high‐risk factors interstitial lung disease, end‐stage chronic obstructive pulmonary disease, patients who have undergone cardiopulmonary resuscitation, acute myocardial ischemia or severe arrhythmia, severe bradycardia, chronic liver or kidney dysfunction, pulmonary embolism, pregnancy, patients aged 70 years or older, patients with malignant tumors, hematological diseases, rheumatic immune system diseases, chronic liver disease, or chronic kidney disease, and patients on long‐term use of immunosuppressants.

At admission, the overall condition of the admitted patients was documented, which included gender, age, primary diagnosis, the APACHE II score, and the Sequential Organ Failure Assessmen (SOFA) score.

### 2.2 Clinical samples

Peripheral blood samples were collected within 24 h of admission using density gradient centrifugation combined with the differential adhesion method to extract mononuclear cells. Immunofluorescence with CD14^+^ antibody (a marker for mononuclear cells) was used to confirm the purity of the isolated mononuclear cells. Total RNA from CD14^+^ cells was extracted using the TRIzol protocol, and quantitative PCR (qPCR) was performed to monitor IBR1 expression.

### 2.3 Mice

The animal experiments were approved by Shandong First Medical University's Animal Care and Use Committee. All procedures followed the Institutional Animal Use and Care Committee's guidelines.

*IBR1^flox/flox^Lyz2‐cre (IBR1^−/−^)* mice, *IFIH1^flox/flox^Lyz2‐cre (IFIH1^−/−^)* mice, and *wild‐type (WT)* mice were obtained from Biomodel Organisms. Male C57BL/6 mice aged 6‒8 weeks were kept at the Laboratory Animal Center in Jinan, China. Genotyping was performed using PCR to confirm the target genotypes. The knockout (KO) efficiency in bone marrow‐derived macrophages (BMDMs) from *IBR1^−/−^*, *IFIH1^−/−^* and *WT* mice was evaluated using PCR and Western blot analysis.

### 2.4 ARDS mouse models

The mice were anaesthetised by intraperitoneal injection of 5.0% pentobarbital sodium solution at a dosage of 4.0 mL/kg of body weight. They were subsequently given intratracheal injections of LPS (15 mg/kg), *Klebsiella pneumoniae* (60 × 10^8^ CFU/kg), IBR1 oligo (2 mg/kg), or polyinosinic‐polycytidylic acid (poly(I:C); 2 mg/kg) 24 h prior to inducing ARDS. Placebo procedures were carried out in a comparable fashion, utilising equal amounts of PBS. Each experimental group consisted of six mice.

Bronchoalveolar lavage fluid (BALF) specimens were collected by exposing the trachea via neck dissection for intubation, followed by three lung lavages with .8 mL PBS. The recovered fluid was centrifuged at 1500 rpm for 10 min at 4°C. The supernatant was stored at −20°C for cytokine detection, while the cell pellet was suspended in 2 mL PBS with 1% bovine serum albumin for flow cytometry analysis.

Pathological lung tissue samples from the experimental groups were extracted, sectioned and prepared for haematoxylin and eosin (H&E) and immunohistochemical staining.

### 2.5 Evans blue permeability assay

Mice were injected with Evans Blue dye and euthanised. Lung tissues were harvested, processed and photographed. Evans Blue solution was prepared in saline at various concentrations. Absorbance was measured to create a standard curve. Lung tissues were minced and incubated in formamide. Supernatant was collected and absorbance measured to determine Evans Blue content, reflecting microvascular permeability.

### 2.6 IFIH1‐M‐EVs treatment

In order to neutralise endogenous and exogenous dsRNA in ARDS lungs and attenuate dsRNA‐mediated pulmonary inflammatory injury, we administered IFIH1‐M‐EVs via intravenous injection for the treatment of LPS‐ARDS, with blank‐EVs serving as the treatment control group. RAW264.7 cells were transfected with either IFIH1‐M or blank plasmids to collect exosomes, following the detailed protocol described in our previous study.

To validate the successful construction of IFIH1‐M‐EVs, we employed NTA and representative electron micrographs of extracellular vesicles (EVs) isolated from the conditioned medium of RAW264.7 macrophages. Western blot analysis showed the presence of characteristic EV surface marker proteins (Alix, CD9 and CD63), and the absence of the negative marker protein (GM130) in the EV samples. Additionally, the presence of Flag‐IFIH1‐M protein was observed in EVs derived from the Flag‐IFIH1‐M protein‐transfected group.

Following the establishment of the LPS‐induced ARDS model, IFIH1‐M‐EVs or blank‐EVs were immediately intravenously injected at a dosage of 1 × 10^10^ particles/mouse. Lung tissues and BALF were collected 24 h later for analysis.

### 2.7 Distribution of EVs

To evaluate the in vivo tissue distribution of IFIH1‐M‐EVs, mice were intravenously administered DiD‐labelled EVs (1 × 10^10^ particles/mouse) or an equivalent volume of PBS. Mice were euthanised within 24 h after injection, and major organs including the heart, lungs, liver, kidneys and spleen were collected. The protocol for labelling EVs with DiD is detailed in our prior investigation.

### 2.8 Lung histopathology

Lung histopathology was assessed by sectioning the right upper lobe into five 5‐µm thick slices after paraffin embedding. These slices were stained with H&E. Histopathological features such as oedema, inflammation, bleeding, atelectasis, necrosis and hyaline membrane formation were scored from 0 to 4. The total score indicated the degree of lung injury. Additionally, the lung wet weight to body weight ratio was measured to assess pulmonary oedema.

### 2.9 Cells

For the isolation and differentiation of BMDMs, mice were euthanised via cervical dislocation. The femurs and tibias were cleaned with 75% ethanol for 30 min. The ends of the bones were cut, and the bone marrow was extracted using a 10 mL syringe with culture medium. The cell suspension collected was centrifuged at 1000 rpm, followed by resuspending the pellet in fresh medium. The isolated cells were incubated in Dulbecco's modified Eagle medium (DMEM) containing 10% foetal bovine serum (FBS) and 20 ng/mL macrophage colony‐stimulating factor for 7 days to promote their differentiation into adherent cells known as BMDMs.

The isolation, differentiation and identification of CD14^+^ mononuclear cells peripheral blood samples were obtained from healthy donor. Mononuclear cells were separated by utilising density gradient centrifugation and the differential adhesion technique. Immunofluorescence staining with a CD14^+^ antibody, a marker for mononuclear cells, was performed to verify the purity of the isolated mononuclear cells. The CD14^+^ mononuclear cells that were isolated were promptly utilised for subsequent experiments.

Naïve CD4^+^ T‐cell isolation: euthanise mice by cervical dislocation, remove the spleen and place it in a dish with diluent. In a laminar flow hood, optionally rinse with PBS, grind spleen fragments through a filter, and collect the cell suspension. Centrifuge at 1500 rpm for 5 min, discard the supernatant, resuspend in PBS, layer over lymphocyte separation solution and centrifuge at 700 g for 20 min. Aspirate the middle layer, wash with cell washing solution and centrifuge at 250 g for 10 min. Resuspend 10^7^ cells in 40 µL buffer, add 10 µL biotin‐antibody cocktail, incubate at 4°C for 5 min, then add 20 µL Anti‐Biotin MicroBeads and 10 µL CD44 MicroBeads and incubate for 10 min. Wash cells with 2 mL PBS, centrifuge, discard supernatant, resuspend in 3 mL PBS, and use MiniMACS Separator with LS column to isolate cells, washing with PBS and collecting the eluted fraction. Centrifuge, discard supernatant, resuspend cells in 1640 medium with 10% FBS, and seed for further experiments.

BMDMs, CD14^+^ mononuclear cells and naïve CD4^+^ T cells were cultured in DMEM with 10% FBS, 100 IU/mL penicillin and 100 µg/mL streptomycin.

### 2.10 Plasmids

The IBR1, IFIH1 and IFIH1 mutant plasmids used in this study were obtained from Zhongchu Biotechnology Company. The cloning primers utilised for the construction of these plasmids can be found in Table S3. The IFIH1‐WT‐Flag (full‐length), IFIH1‐M1‐Flag (7−190), IFIH1‐M2‐Flag (317−872) and IFIH1‐M2‐Flag (893−1020) plasmids were created by incorporating the respective DNA fragments into the pET28a vector through the process of homologous recombination. The desired DNA fragments, including IFIH1‐WT, IFIH1 mutants and Flag tag, were amplified using the PCR technique. The amplified fragments were then ligated to the linearised pET28a vector using the Gibson Assembly kit. To ensure the accuracy and fidelity of the plasmid constructs, Sanger sequencing was performed to confirm the integrity of the DNA sequences.

### 2.11 Reagents

LPS: *Escherichia coli* 0111:B4‐derived LPS (Sigma‒Aldrich; catalogue number L2630) was employed. LPS concentration was established from previous studies, set at 500 ng/mL for in vitro experiments and 15 mg/kg for the LPS‐induced mouse model.

KP: *K. pneumoniae* (SHBCC D11105 CMCC46117; Shanghai Bioresource Collection Center) was utilised to induce the ARDS model. The concentration of KP was determined based on our previous research, established at 60 × 10^8^ CFU/kg.

Poly(I:C): poly(I:C) (MedChemExpress; catalogue number HY‐107202) was utilised in the in vitro experiments. The concentration of poly(I:C) was established at 10 ng/mL, according to findings from our previous studies.

Purified proteins: the IFIH1‐WT‐Flag (full‐length), IFIH1‐M1‐Flag (7−190), IFIH1‐M2‐Flag (317−872) and IFIH1‐M2‐Flag (893−1020) were purchased from Zhongchu Biotechnology Company. These proteins were overexpressed and truncated in BL21 (DE3)‐competent *E. coli* cells. The His‐tag systems were employed for the purification of these proteins. The purity and integrity of the purified proteins were confirmed using Coomassie staining.

Synthetic RNAs: synthetic RNAs corresponding to IBR1‐WT and its truncated mutants were purchased from Sangon Biotech. These RNAs were generated using T7 in vitro transcription based on their respective sequences, as depicted in Table S4. Biotin labelling was applied to accurately label the 5′ and 3′ ends of IBR1‐WT. The concentration of IBR1 was determined to be 10 ng/mL for in vitro experiments and 2 mg/kg for in vivo experiments.

### 2.12 Western blot

Western blotting involved SDS‐PAGE for protein separation and transfer to polyvinylidene fluoride (PVDF) membranes. Membranes were incubated with primary antibodies (1:1000) overnight at 4°C, followed by secondary antibodies for 1 h at room temperature. Proteins were detected using enhanced chemiluminescence. To guarantee precise normalisation of protein expression levels, the expression levels of β‐tubulin were employed as loading controls.

Table S5 displays the data on antibodies.

### 2.13 RT‐PCR

Total RNA was extracted from lung tissue samples or cells using TRIzol reagent following the manufacturer's protocol. Reverse transcription of RNA was performed using PrimeScript Reverse Transcription Mix. RT‐PCR was conducted using SYBR Premix Ex Taq and a Step One Plus RT‐PCR system according to the manufacturer's instructions. The levels of expression were standardised with the reference gene, β‐actin and measured using the 2^ΔΔCt^ technique.

Agarose gel electrophoresis was used to visualise genomic DNA/RNA fragments from transgenic mouse tail snips, RNA immunoprecipitation (RIP) and macrophages. The primer sequences are listed in Table S6.

### 2.14 Flow cytometry

BALF and BMDMs were collected from mice in this study. To block non‐specific binding, the cells were incubated with a blocking solution for 20 min. Subsequently, the cells were labelled with APC‐conjugated anti‐mouse F4/80 (1:200) and/or PE‐conjugated anti‐mouse CD86 (1:200) antibodies, as well as FITC‐conjugated anti‐mouse CD206 (1:200), which target macrophages and M1/M2 macrophages, respectively. The fluorescence intensity of CD86 and CD206 was measured to assess their expression levels. Flow cytometry analysis was performed using an ACEA NovoCyte flow cytometer (ACEA Biosciences) and data were acquired and analysed using Novo Express (ACEA Biosciences) and FlowJo X (Tree Star) software, respectively. The data on antibodies are displayed in Table S5.

### 2.15 Dot plots

Poly(I:C), IBR1‐WT and IBR1 mutation samples were adjusted in nuclease‐free water to concentrations of 10, 5 and 1 ng/mL, respectively. Then, 5 µL aliquots of each sample were spotted onto a positively charged nylon membrane (Whatman NytranSuPerCharge, Sigma‒Aldrich).

The membrane was probed with J2 anti‐dsRNA murine antibody at a 1:1000 dilution for dsRNA detection. Chemiluminescence was performed using Amersham ECL Prime Western Blotting Detection Reagent and imaged with the ChemiDoc MP Imaging System.

J2: Abcam, rabbit mAb, # ab288755.

### 2.16 Enzyme‐linked immunosorbent assay

Pro‐inflammatory cytokines such as interleukin‐1 beta (IL‐1β), chemokine (C‒C motif) ligand 2 (CCL2), tumour necrosis factor alpha (TNF‐α) and interleukin‐6 (IL‐6) levels were measured in macrophage culture supernatant and BALF using enzyme‐linked immunosorbent assay (ELISA) kits.

The specific ELISA kits used for each cytokine were as follows:

IL‐1β: MLB00C, R&D Systems;
CCL2: MJE00B, R&D Systems;
TNF‐α: MTA00B, R&D Systems;
IL‐6: M6000B, R&D Systems.

### 2.17 Immunohistochemistry

The lung tissue slices underwent deparaffinisation and were then incubated with 5% goat serum. Primary antibodies (diluted 1:100) were incubated overnight at 4°C. After rinsing with PBS, a biotinylated secondary antibody (1:50 dilution) was applied. The ImageJ software was used to quantify the positive areas, while photographs were captured using an Olympus light microscope at a magnification of 400×.

### 2.18 RNA fluorescence in situ hybridisation

Fluorescence in situ hybridisation (FISH) was conducted on BMDMs or CD14^+^ monocytes utilising the Ribo Fluorescent In Situ Hybridisation Kit from Ribo Bio. The cells underwent a brief wash with PBS followed by fixation in 4% formaldehyde at room temperature for 15 min. Cells were permeabilised in PBS with .5% Triton X‐100 on ice for 5 min. After PBS washes, cells were blocked with hybridisation solution at room temperature for 30 min.

The hybridisation was performed with the IBR1 FISH Probe Mix in a humidified chamber at 37°C for 12−16 h. For co‐localisation studies, cells underwent RNA FISH and IFIH1 antibody labelling, then were fixed for 5 min in 2% formaldehyde. Immunofluorescence was then performed using an anti‐DOT1L primary antibody, followed by application of fluorescent secondary antibodies. 4, 6‐Diamidino‐2‐phenylindole, dihydrochloride (DAPI) was used to counterstain the cell nuclei.

IFIH1: Invitrogen, rabbit mAb # 33H12L34;
CD14: Proteintech, rabbit pAb # 17000‐1‐AP;
IBR1‐mus probe: GCCCTGTAGTTGCTGTAGTCCC, 5′CY3 modification

### 2.19 Immunoblotting and RNA immunoprecipitation

To investigate the interaction between IBR1, IFIH1 and IFIH1 mutant proteins and RNA, cultured BMDMs were transfected with IBR1, IFIH1 and IFIH1 mutant plasmids for 24 h. The cells were collected with .5 mL of lysis buffer following the Magna RIPTM RNA‐binding protein immunoprecipitation kit (17‐700, Millipore) instructions. Magnetic beads were washed, and 5 µg of the specific antibody was added to the beads in RIP washing buffer and then incubated at room temperature for 30 min. The RIP lysate was combined with the antibody‐bound beads in RIP immunoprecipitation buffer and incubated overnight at 4°C. After removing the supernatant, the pellet was resuspended in immunoprecipitation buffer with proteinase K and incubated at 55°C for 30 min with shaking to digest the proteins. The purified RNA was analysed for IBR expression by PCR, with IgG as a negative control to verify specificity.

### 2.20 RNA pull‐down assay

To study the interaction between IBR1 and its binding proteins, BMDMs were lysed in 500 µL of co‐immunoprecipitation buffer. The lysates were incubated with 6 µg of biotin‐labelled IBR1 probe at room temperature for 2 h. Then, 50 µL of washed Streptavidin C1 magnetic beads were added and incubated for another hour at room temperature. The proteins bound in the pull‐down materials were examined through silver staining and Western blotting methods. The bound proteins in the pull‐down materials were analysed using silver staining and Western blot techniques.

### 2.21 RNA electrophoretic mobility shift assay

Electrophoretic mobility shift assay (EMSA) was performed using 5′ and 3′‐biotin‐labelled IBR1. Unless noted, .1 pmol of biotin‐labelled IBR1 was combined with 30 pmol of purified IFIH1 or its mutant in buffer A (20 mM N‐2‐hydroxyethylpiperazine‐N‐ethane‐sulphonicacid (HEPES), pH 7.5, 150 mM NaCl, 1.5 mM MgCl_2_ and 2 mM Dithiothreitol (DTT)) with 2 mM adenosine diphosphate phosphocreatine (ADPCP). The mixture was incubated for 10 min at room temperature and then analysed using BisTris native gels. For time‐dependent EMSA, the samples were incubated at a temperature of 37°C for the specified duration.

### 2.22 RNA sequencing and RIP sequencing

Total RNA was extracted from BMDMs and CD14^+^ monocytes using TRIzol. Following rRNA depletion, the residual RNA was processed using Illumina's guidelines for library preparation.

For RIP sequencing (RIP‐seq), RNA extracts from BMDMs were subjected to RNA‐binding protein immunoprecipitation using the Magna RIPTM kit. The IFIH1‐bound RNA complexes were enriched using IFIH1 antibody‐bound beads and RIP immunoprecipitation buffer. The purified RNAs were then sequenced using Illumina Sequencing Technology, with 5% input RNA as a negative control to reduce noise. Sequences of New Transcripts are shown in Table S7.

RNA sequencing (RNA‐seq) and RIP‐seq datasets generated in this study have been archived in the GEO database Home—GEO—NCBI (nih.gov) under the accession codes GSE244502, GSE247176, GSE247177 and GSE245440.

### 2.23 Statistical analyses

All data are presented as mean ± standard error of the mean unless otherwise indicated. Data normality was evaluated with Shapiro‒Wilk tests. For skewed data, log transformation was used. Statistical significance was assessed using unpaired Student's *t*‐test, Welch's *t*‐test, or one‐ or two‐way analysis of variance as appropriate.

In the in vivo experiments, there were six mice per group, whereas in the in vitro experiments, there were three mice per group. Histology analysis and Western blot analysis were independently repeated three times with consistent results. A *p*‐value below .05 was deemed to be statistically significant. A statistically significant finding was defined as having a false discovery rate (FDR)‐adjusted *p*‐value below .05 (FDR‐adjusted *p* < .05).

The statistical analyses in current study were conducted using R version x64 4.1.1.

## 3 RESULTS

### 3.1 IBR1 and IBR2 are novel transcripts that interact with IFIH1

**FIGURE 1:**
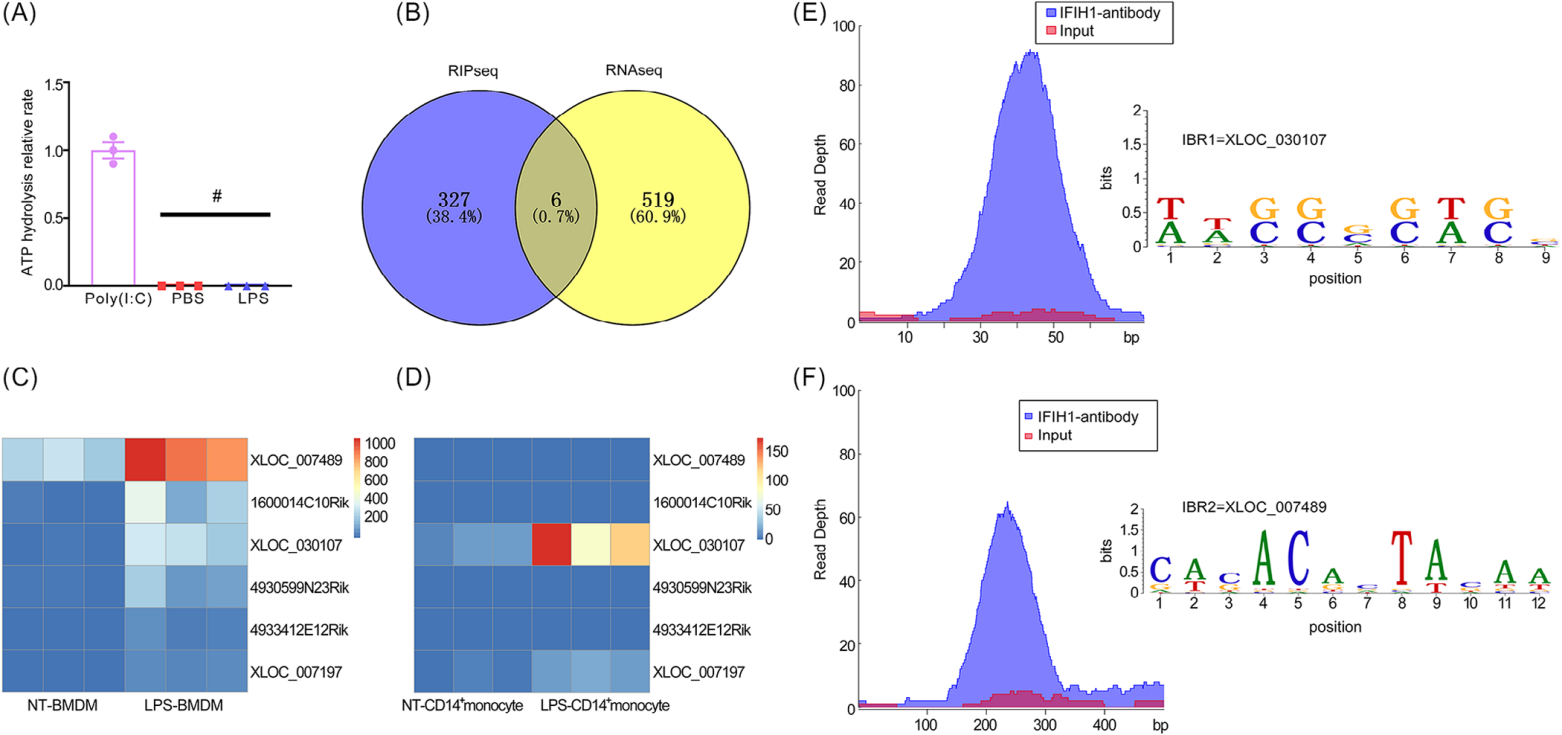
High expression levels of the IFIH1‐binding RNA molecules IBR1 and IBR2 in lipopolysaccharide (LPS)‐treated monocytes/macrophages from human and mouse sources. (A) Comparison of the ATP hydrolysis rates of IFIH1 in the presence and absence of LPS (500 ng/mL) relative to those in the presence of polyinosinic‐polycytidylic acid (poly(I:C)) (10 ng/mL). ATP hydrolysis is an indicator of IFIH1 activation. (B) Venn diagram analysis showing the overlap of the RIP sequencing (RIP‐seq) peaks in the LPS‐treated bone marrow‐derived macrophages (BMDMs) and the RNA‐seq peaks in the non‐treated (NT) and LPS‐treated BMDMs. Six RNA candidates were identified in the overlap that were IFIH1‐binding RNAs and highly expressed in LPS‐treated BMDMs (false discovery rate [FDR] <.05, log‐fold change [log FC] ≥2). (C) Heatmap depicting the expression levels of the six RNA candidates in NT and LPS‐treated BMDMs on the basis of RNA‐seq data. The RNA expression levels are presented as counts obtained from RNA‐seq. (D) Heatmap showing the expression levels of the six RNA candidates in NT and LPS‐treated CD14^+^ monocytes from healthy donors, as determined from the RNA‐seq data. The RNA expression levels are presented as counts obtained from RNA‐seq analysis. (E and F) Genome browser visualisation of RIP‐seq peaks for IFIH1 at the IBR1 and IBR2 regions in BMDMs treated with LPS.

IFIH1 is a highly conserved cytoplasmic viral RNA sensor that detects dsRNA from various viruses and exogenous RNA. The activation of IFIH1 is characterised by ATP hydrolysis.^11^, ^12^, ^13^ Previous studies have demonstrated that IFIH1 can be activated by LPS in macrophages.^8^ To determine whether the activation of IFIH1 by LPS is direct or indirect, we performed an in vitro experiment utilising purified recombinant IFIH1 derived from *E. coli* to assess ATP hydrolysis rates. Poly(I:C), a synthesised dsRNA, was utilised as the positive control. We observed a significant increase in the ATP hydrolysis activity of IFIH1 upon stimulation with poly(I:C) but not with LPS (Figure 1A). These results indicated that LPS triggers IFIH1 activation in macrophages via an indirect pathway.

To elucidate the molecular mechanism underlying LPS‐induced IFIH1 activation, we used RNA‐seq and RIP‐seq techniques. Venn diagram analysis was performed to assess the overlap between the RIP‐seq peaks in the LPS‐treated BMDMs and the RNA‐seq peaks in non‐treated and LPS‐treated BMDMs. We identified six RNA candidates exhibiting the dual characteristics of binding to IFIH1 and displaying high expression levels in LPS‐treated BMDMs, meeting the stringent statistical criteria of FDR‐adjusted *p* < .05 and log‐fold change (log FC ≥2), as depicted in Figure 1B. Among these candidates, three were known RNAs (1600014C10Rik, 4930599N23Rik and 4933412E12Rik), whereas three were novel transcripts (XLOC_030107, XLOC_007489 and XLOC_007197). The expression levels of these RNAs were determined by analysing the RNA‐seq counts (Figure 1C).

To establish the clinical relevance of the identified candidates, we performed homology verification between humans and mice by conducting RNA‐seq on CD14^+^ human monocytes. A library was built using the sequences of the six candidates as templates. The results of sequence alignment from RNA‐seq of CD14^+^ monocytes revealed that only XLOC_030107 and XLOC_007197 were upregulated in LPS‐induced CD14^+^ monocytes compared with non‐treated CD14^+^ monocytes, with an FDR < .05, as shown in Figure 1D. Given that these two transcripts were found to bind to the IFIH1 protein, we designated XLOC_030107 as IFIH1‐binding RNA 1 (IBR1) and XLOC_007197 as IFIH1‐binding RNA 2 (IBR2). The binding sites and peaks of IFIH1 in the IBR1 and IBR2 regions are shown in Figure 1E,F.

### 3.2 IBR1 directly activates IFIH1 and is upregulated in LPS‐induced macrophages/monocytes

**FIGURE 2:**
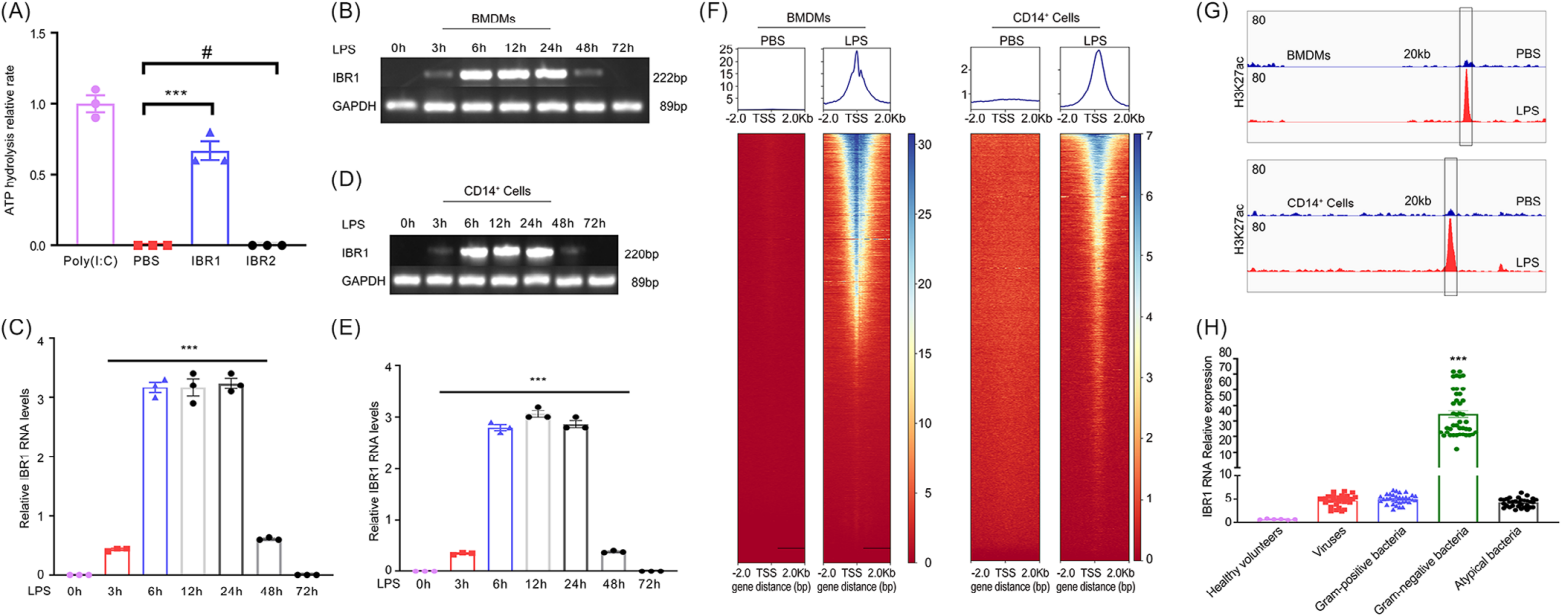
Endogenous activator IFIH1‐binding RNA 1 (IBR1): highly expressed in CD14^+^ monocytes from acute respiratory distress syndrome (ARDS) patients with Gram‐positive bacterial pneumonia. (A) The ATP hydrolysis rates of IFIH1 were compared with or without IBR1 (10 ng/mL) and IBR2 (10 ng/mL) relative to the rates with polyinosinic‐polycytidylic acid (poly(I:C)) (10 ng/mL). (B‒E) PCR analysis was conducted to assess the relative expression of IBR1 RNA in bone marrow‐derived macrophages (BMDMs) and CD14^+^ monocytes upon stimulation with lipopolysaccharide (LPS) (500 ng/mL) at various time points. The results demonstrated that IBR1 was upregulated at 3 h after LPS stimulation, reached its peak at 6−24 h and subsequently started to decrease at 48 h. (F) Assessing chromatin accessibility: ATACseq heatmaps and peak charts were utilised to evaluate chromatin region dynamics, with a specific focus on IBR1 and its promoter region, in macrophage polarisation. (G) Comparative analysis of ChIP tag density profiles at IBR1 and its promoter region for mouse and human H3K27ac. (H) qPCR analysis was performed to examine the expression of IBR1 RNA in CD14^+^ monocytes obtained from six healthy donors and 140 patients diagnosed with ARDS. The patients were categorised on the basis of the type of infection, including respiratory viruses (33 cases), Gram‐positive bacteria (33 cases), Gram‐negative bacteria (42 cases) and atypical bacteria (32 cases). The presented data represent the mean ± standard deviation, and statistical significance was determined by appropriate statistical tests (^*^ *p *< .05, ^**^ *p *< .01, ^***^ *p *< .001).

To investigate the potential activation of IFIH1 by IBR1 and IBR2, we evaluated the ATP hydrolysis rates of IFIH1 with IBR1 and IBR2 oligos prepared via T7 in vitro transcription. Our findings revealed that IBR1 induced ATP hydrolysis of IFIH1, whereas IBR2 did not, as illustrated in Figure 2A. The detailed sequences and relevant information regarding IBR1 are provided in Table S1. These results indicated that IBR1 can directly activate the IFIH1 protein, whereas IBR2 cannot.

We performed PCR to evaluate the expression profile of IBR1 in LPS‐induced BMDMs/CD14^+^ monocytes at different time intervals. The isolation and verification of CD14^+^ monocytes were performed via immunofluorescence microscopy (Figure S1). BMDMs were isolated and verified through flow cytometry analysis (Figure S2).

The results demonstrated that IBR1 was upregulated at 3 h after LPS stimulation, reached its peak at 6−24 h, and subsequently started to decrease at 48 h (Figure 2B‒E). The results indicated that the expression of IBR1 at 6, 12 and 24 h induced by LPS was significantly greater than that at 0 and 72 h, with *p* < .001.

To explore the mechanism underlying the upregulation induced by LPS, we utilised ATAC‐seq to assess the chromatin accessibility of the IBR1 gene and conducted ChIP‐seq using an H3K27ac antibody to evaluate the activity of the IBR1 promoter and enhancer regions (Figure 2F,G). These results suggested that LPS induces chromatin accessibility opening of IBR1 and activates both the IBR1 promoter and the enhancer.

Additionally, the top 20 differentially expressed genes and the pathway analysis comparing LPS‐treated BMDMs and PBS‐treated BMDMs are presented in Figure S3.

These findings suggested that IBR1 is a novel transcript that binds to IFIH1 and may contribute to regulating IFIH1‐mediated immune responses.

### 3.3 IBR1 upregulation in patients with Gram‐negative bacteria‐induced ARDS

To investigate the relationships among IBR1, LPS and ARDS, we conducted a study with six healthy volunteers and 140 patients with ARDS caused by pneumonia. Among these patients, 33 were infected with respiratory viruses, 33 with Gram‐positive bacteria, 42 with Gram‐negative bacteria and 32 with atypical bacteria. The detailed clinical profiles of the patients are presented in Table S2. qPCR was subsequently used to measure the levels of IBR1 in monocytes.

The results revealed significant upregulation of IBR1 in patients with Gram‐negative bacteria‐induced ARDS compared with both healthy volunteers and other ARDS patient groups (*p* < .001, Figure 2H). This finding indicated that IBR1 may contribute specifically to the development of ARDS caused by Gram‐negative bacterial infections.

### 3.4 IBR1 binding to IFIH1

**FIGURE 3:**
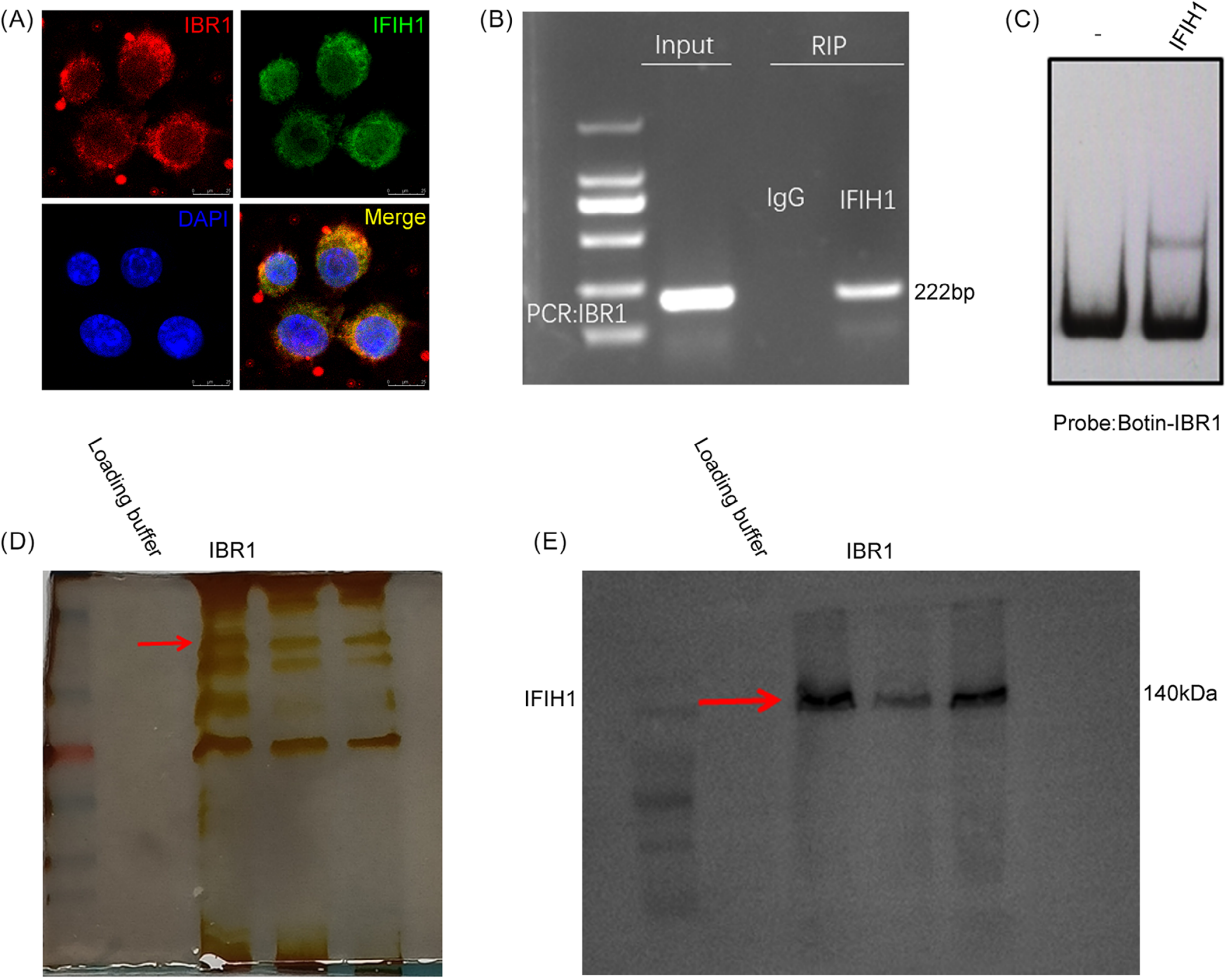
Binding of IFIH1‐binding RNA 1 (IBR1) to IFIH1. (A) RNA fluorescence in situ hybridisation (FISH) and immunofluorescence (IF) experiments revealed that IBR1 co‐localised with IFIH1 in bone marrow‐derived macrophages (BMDMs). Scale bar: 25 µm. (B) RNA immunoprecipitation (RIP)‒PCR detection of the enrichment of IFIH1 on IBR1 in BMDMs. Immunoglobulin G (IgG) was used as the negative control. (C) Electrophoretic mobility shift assays (EMSAs) revealed that IBR1 binds to IFIH1 in vitro. (D and E) Silver staining of gels after sodium dodecyl sulfate polyacrylamide gel electrophoresis (SDS‒PAGE) and Western blotting revealed that IFIH1 was enriched, as determined by IBR1 pull‐down assays.

To investigate the mechanism by which IBR1 influences macrophage M1 polarisation and IFIH1 activation, we performed RIP‐seq analysis and found that IBR1 bound to IFIH1, potentially leading to its activation. To validate this hypothesis, we conducted FISH, RNA pull‐down assays, RIP‒PCR and EMSAs to identify RNA‒protein interactions. The results of the RNA FISH experiments confirmed the co‐localisation of IBR1 and IFIH1 in the cytoplasm of BMDMs (Figure 3A). The specificity of the RNA‒protein interaction was validated by RIP (Figure 3B).

Additionally, we produced purified IFIH1 protein, synthesised IBR1 via T7 transcription, and then conducted EMSA experiments in Eppendorf tubes without a cellular environment. The EMSA results indicated that IBR1 bound to the IFIH1 protein. Furthermore, we found that in naïve CD4^+^ T cells, IBR1 also bound to IFIH1 (Figure S4). These results suggested that the interaction between the IBR1 and IFIH1 proteins is not cell specific. Finally, RNA pull‐down assays followed by Western blotting revealed a strong interaction between IBR1 and IFIH1 (Figure 3C‒E).

### 3.5 IBR1 binds specifically to the helicase domain of IFIH1

**FIGURE 4:**
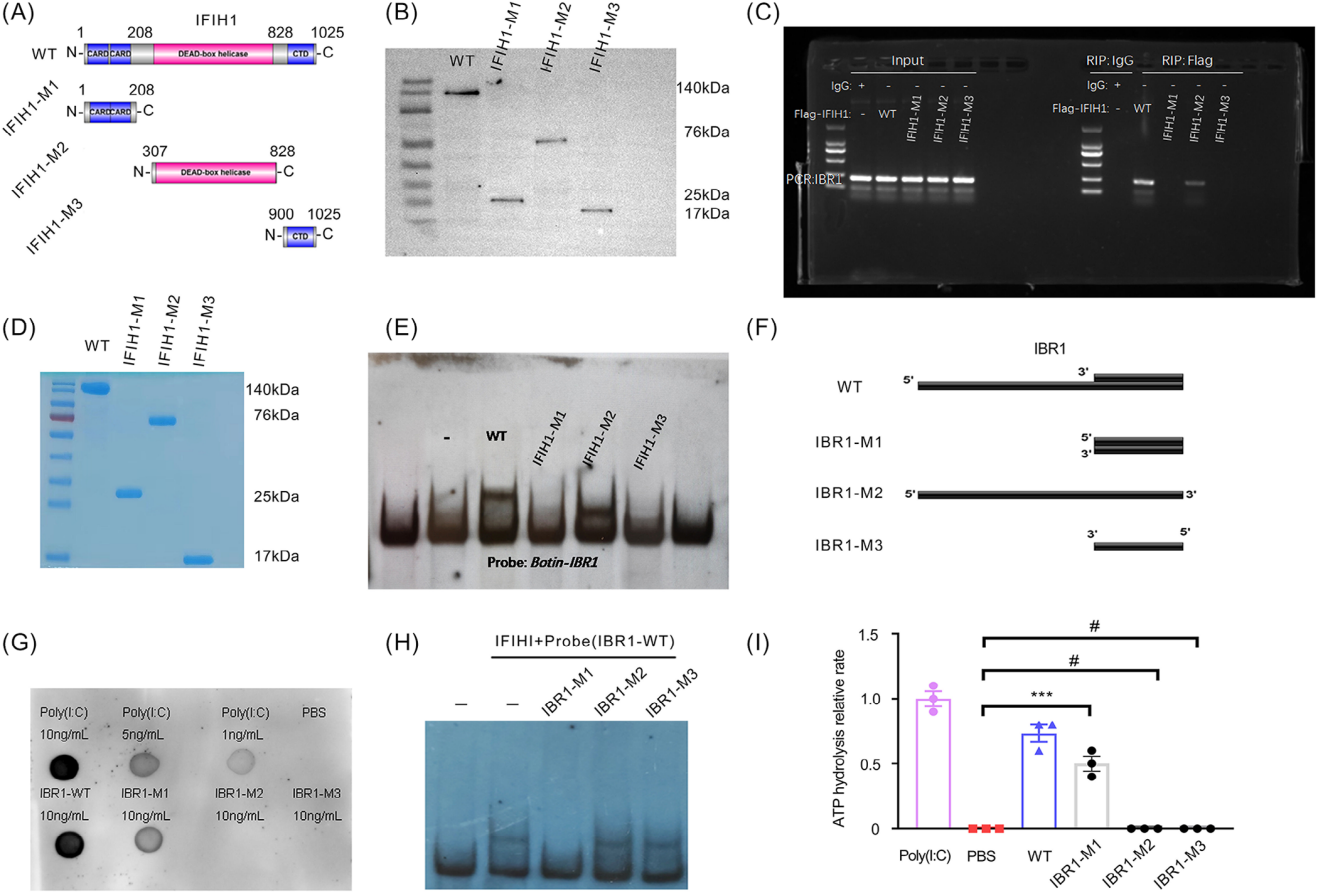
Binding of IFIH1‐binding RNA 1 (IBR1) to the IFIH1 helicase domain via local duplex structure formation. (A) Schematic representation of wild‐type (WT) and truncated mutants (M) of IFIH1. (B) In bone marrow‐derived macrophages (BMDMs), cotransfection of Flag‐tagged IFIH1‐WT and truncated mutant plasmids with IBR1‐WT plasmids resulted in the expression of IFIH1‐WT and mutant proteins, as confirmed by Western blotting. (C) RNA immunoprecipitation (RIP) assay showing the binding of IBR1 to Flag‐IFIH1‐WT or its truncation mutants in BMDMs. (D) Overexpression and purification of WT and truncation mutants of IFIH1 in *Escherichia coli*, as confirmed by Coomassie blue staining. (E) Electrophoretic mobility shift assay (EMSA) demonstrating the binding of the helicase domain (amino acids 307‒828) of IFIH1 to biotin‐labelled IBR1‐WT. (F) A diagram illustrating the WT and IBR1 truncation mutants. (G) Dot blot analysis was conducted with the J2 mAb to detect the presence of double‐stranded RNA (dsRNA) formation in aliquots of IBR1 WT and truncated mutant oligos. (H) The specific recognition of the formation of local duplex structures that bind to IFIH1 in IBR1 was investigated by EMSA. Biotin‐labelled IBR1‐WT (1 pmol) was incubated with full‐length IFIH1 (1‒1025, 30 pmol) in the EMSA experiment. The cold RNAs of IBR1 mutants (50 pmol) were used as competitive inhibitors. (I) The ATP hydrolysis rates of IFIH1 were compared in the presence of IBR1 WT and the truncated mutant oligos (at a concentration of 10 ng/mL) relative to the rates with poly(I:C) (at a concentration of 10 ng/mL).

To determine the specific interaction between IBR1 and IFIH1, we sought to identify the domains of IFIH1 that are responsible for this interaction. Previous research has identified three distinct functional domains within the IFIH1 CARD (amino acids 7−190), helicase domain (amino acids 317−872) and CTD (amino acids 893−1020). To investigate the domains involved, we constructed plasmids expressing Flag‐tagged IFIH1‐WT and functional domain‐truncated mutants (Figure 4A) and cotransfected them with IBR1‐WT plasmids into BMDMs. Western blot analysis with an anti‐Flag antibody validated the successful expression of IFIH1‐WT and the truncated mutant proteins containing the functional domain in BMDMs (Figure 4B). We subsequently performed a Flag RIP‒PCR assay, which revealed that IBR1 failed to bind to the Flag‐IFIH1‐M1 and M3 mutant proteins (helicase domain deletion), indicating the essential role of the helicase domain in the interaction between IBR1 and IFIH1 (Figure 4C).

To further validate these findings in vitro, we overexpressed WT and truncated mutants of IFIH1 in BL21 (DE3)‐competent *E. coli* cells and purified the proteins. The purity and integrity of the purified proteins were confirmed by Coomassie blue staining (Figure 4D). An in vitro EMSA was then performed by incubating biotin‐labelled IBR1‐WT (.1 pmol) with either the WT or the truncated mutants of IFIH1 (30 pmol). The EMSA results further confirmed that IBR1 specifically bound to the helicase domain of IFIH1 and not to other domains (Figure 4E). These results provided valuable information about the molecular foundation of the interaction between IBR1 and IFIH1, emphasising the crucial function of the helicase domain in facilitating this interaction.

### 3.6 IBR1 interacts with IFIH1 through the creation of local duplex structures

Notably, the recognition of viral RNA by IFIH1 is not dependent on the 5′ triphosphate or blunt ends but rather relies on the formation of duplex structures.^11^, ^12^, ^13^ Through motif analysis of RIP‐seq data for IFIH1, we identified potential double‐stranded complementary structures between the domains of IBR1 and IFIH1. Furthermore, the 5′ end of the IBR1 sequence contains multiple complementary bases (TAAGGGCTGCGCTGGAAC‐GTTCCAGCGCAGCCCTTA). Previous studies have shown that this RNA 5′ terminal sequence is prone to folding inwards, resulting in the formation of double‐stranded structures known as RNA hairpins. The mechanism of hairpin formation is likely related to the rebinding of RNA polymerase to the 5′ end of the transcript after transcription is completed.

To confirm our hypothesis, we carried out a series of experiments. First, we generated IBR1 and truncated mutant RNAs by T7 in vitro transcription on the basis of their respective sequences, as depicted in Figure 4F and Table S5. Biotin labelling was applied to accurately label the 5′ and 3′ ends of IBR1‐WT. Subsequently, we performed dot blot analysis using the J2 monoclonal antibody to detect the presence of dsRNA formation in aliquots of IBR1‐WT and truncated mutant oligos. Notably, the dot blot results clearly indicated the absence of interaction between IBR1‐M2 and IBR1‐M3 with the J2 antibody, strongly suggesting the deletion of double‐stranded base sequences (Figure 4G).

To strengthen the evidence of our results, we performed an in vitro EMSA. Biotin‐labelled IBR1‐WT (.1 pmol) was used as a probe, while other mutant RNAs served as competitive inhibitors in the presence of IFIH1‐WT protein (30 pmol). The EMSA results provided compelling evidence to support our hypothesis, as they demonstrated the failure of IBR1‐M2 and IBR1‐M3 to bind to the IFIH1 protein (Figure 4H).

Furthermore, we performed ATP hydrolysis rate experiments to assess the functional implications of IBR1‐WT and the mutant RNAs. Interestingly, our data revealed that IBR1‐M2 and IBR1‐M3 were incapable of activating IFIH1, suggesting that the formation of a double‐stranded structure is indispensable for RNA binding and subsequent activation of IFIH1 (Figure 4I).

These findings significantly contributed to our understanding of the intricate mechanisms underlying the interaction between IBR1 and IFIH1.

### 3.7 IBR1 facilitates LPS‐induced M1 macrophage polarisation and activates IFIH1

To explore the role of IBR1 in the regulation of inflammatory lung injury, M1 macrophage polarisation, and IFIH1 activation in vivo, we generated macrophage‐specific conditional KO mice: *IBR1^−/−^* and *IFIH1^−/−^*. To investigate the role of IBR1 in modulating macrophage M1 polarisation and IFIH1 activation in response to LPS stimulation, we performed KO, rescue and overexpression experiments in BMDMs. IBR1‐KO BMDMs were isolated from *IBR1^−/−^* mice, while rescue IBR1 and IBR1‐overexpressing BMDMs were generated by transfecting overexpression vectors into separate BMDMs from *IBR1^−/−^* and *WT* mice, respectively. Empty vectors were used as blank controls. The mice were genotyped by PCR to ensure that homozygous individuals were obtained (Figure S5). To assess the efficiency of KO, rescue and overexpression, we conducted PCR analysis to measure the RNA expression levels of IBR1 (Figure S6).

**FIGURE 5:**
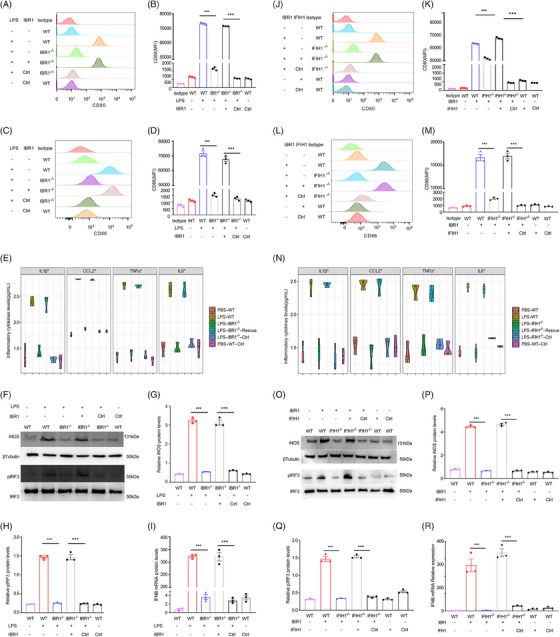
M1 macrophage polarisation mediated by IFIH1‐binding RNA 1 (IBR1) through an IFIH1‐dependent mechanism. (A‒D) Flow cytometric analysis was conducted to measure the production of CD80 and CD86, which are markers of M1 polarisation, by bone marrow‐derived macrophages (BMDMs) isolated from *wild‐type* (*WT*) and *IBR1^−/−^* mice. The cells were stimulated with lipopolysaccharide (LPS) (500 ng/mL) for 24 h with or without IBR1 overexpression. (E) Enzyme‐linked immunosorbent assay (ELISA) was performed to measure the levels of proinflammatory cytokines in the culture medium of BMDMs from the specified experimental groups. (F‒H) Western blot analysis and quantitative analysis were performed to determine the protein levels of iNOS and pIRF3 in BMDMs from the indicated experimental groups. iNOS is a marker of M1 macrophage polarisation, whereas pIRF3 indirectly reflects IFIH1 activation in vivo. (I) qPCR analysis was carried out to investigate the IFNB mRNA level in BMDMs derived from the specified experimental groups. Interferon‐beta (IFN‐β) production indirectly reflects IFIH1 activation in vivo. (J‒M) Flow cytometric analysis was conducted to measure the production of CD80 and CD86 by BMDMs isolated from *WT* and *IFIH1^−/−^* mice. The cells were stimulated with IBR1 oligo (10 ng/mL) for 24 h with or without IFIH1 overexpression. (N) ELISA was performed to measure the levels of proinflammatory cytokines. (O‒Q) Western blot analysis and quantitative analysis were performed to determine the protein levels of iNOS and pIRF3 in the BMDMs. (R) qPCR analysis was performed to investigate the IFNB mRNA level. The presented data represent the mean ± standard deviation, and statistical significance was determined by appropriate statistical tests (^*^ *p *< .05, ^**^ *p *< .01, ^***^ *p *< .001).

Flow cytometry was conducted to assess the levels of CD80 and CD86, key markers indicative of M1 polarisation, in BMDMs derived from *WT* and *IBR1^−/−^* model mice. After stimulation with 500 ng/mL LPS for 24 h with or without IBR1 overexpression, the cells were analysed. The results revealed a significant reduction in CD80 and CD86 expression following IBR1 KO (*p* < .001) (Figure 5A‒D).

ELISAs revealed a notable decrease (*p* < .001) in the levels of IL‐1β, CCL2, TNF‐α, and IL‐6 in the culture medium upon IBR1 deletion (Figure 5E). Furthermore, Western blot analyses revealed a marked decrease (*p* < .001) in LPS‐induced inducible nitric oxide synthase (iNOS) expression in BMDMs from *IBR1^−/−^* mice compared with that in *WT* BMDMs. Moreover, transfection with empty vectors did not affect the expression levels of CD80, CD86 or iNOS (*p* > .05), as shown in Figure 5F,G.

Rescue experiments involving the transfection of IBR1 high‐expression plasmids into *IBR1^−/−^* BMDMs revealed a subsequent increase in iNOS, CD80 and CD86 expression after LPS stimulation, along with comparable alterations in proinflammatory factors. Additionally, a significant increase in the expression of M1‐polarised markers was observed upon IBR1 overexpression in WT BMDMs compared with that observed with control blank vectors (*p* < .001).

To indirectly evaluate the activation of IFIH1 in BMDMs, we examined the expression of pIRF3 and the transcription of IFNB mRNA. Our results revealed a significant reduction in pIRF3 expression and IFNB mRNA transcription after IBR1 KO (*p* < .001, Figure 5H,I). Conversely, overexpression and rescue experiments led to a notable increase in pIRF3 expression and IFNB mRNA transcription compared with those of the control blank vectors (*p* < .001). These findings provided evidence that IBR1 plays a role in regulating the activation of IFIH1.

### 3.8 IBR1: a catalyst for IFIH1 activation and M1 macrophage polarisation

To explore the ability of IBR1 to activate IFIH1 and polarise macrophages towards the M1 phenotype in BMDMs, we treated BMDMs with IBR1 oligo (10 ng/mL) and IBR1 expression vectors (1 ng/mL) for 24 h to stimulate the cells. Compared with the blank controls, both the IBR1 oligo and the IBR1 expression vectors significantly increased the levels of M1‐macrophage protein (iNOS and CD86) and a marker of IFIH1 activation (pIRF3 expression) (*p* < .001), as assessed by flow cytometry and Western blotting (Figure S7). These results provided strong evidence that IBR1 serves as a powerful activator of IFIH1 and enhances the polarisation of macrophages towards the M1 phenotype.

### 3.9 IBR1 elicits M1 macrophage polarisation via an IFIH1‐dependent mechanism

Our findings confirmed that IBR1 functioned as a strong stimulator of M1 macrophage polarisation. To determine whether the effect of IBR1 on macrophage polarisation is dependent on IFIH1, we conducted KO, rescue and overexpression experiments in BMDMs from *IFIH1^−/−^* and *WT* mice with or without plasmid IBR1 expression vectors. The validity of the KO, rescue and overexpression efficiency was confirmed through Western blot analysis (Figures S8 and S9).

The expression of M1‐polarised markers and inflammatory cytokines was evaluated through Western blot, flow cytometry and ELISA methods. We observed significant downregulation of these markers in IBR1 oligo (10 ng/mL, 24 h)‐stimulated BMDMs following IFIH1 silencing (*p* < .001). Moreover, rescue and overexpression experiments demonstrated that restoring IFIH1 expression significantly increased the expression of M1‐polarised markers compared with that observed with control blank vectors (*p* < .001, Figure 5J‒P). Notably, vector transfection did not alter iNOS, CD80 or CD86 levels (*p* > .05).

Furthermore, IFIH1 KO significantly decreased pIRF3 expression and IFNB mRNA transcription in BMDMs (*p* < .001), whereas rescue and overexpression of IFIH1 in BMDMs markedly upregulated pIRF3 and IFNB mRNA transcription (*p* < .001). The above results are shown in Figure 5O‒R.

Taken together, these results strongly suggested that IBR1 triggers M1 polarisation of macrophages via an IFIH1‐dependent pathway.

### 3.10 IBR1 has no effect on the expression of IFIH1

To clarify whether IBR1 regulates the expression of the IFIH1 protein, we treated BMDMs with IBR1 oligonucleotides (10 ng/mL) and IBR1 expression vectors (1 ng/mL) for 24 h. The results of the Western blot analysis indicated that compared with the blank controls, neither the IBR1 oligonucleotides nor the plasmid IBR1 expression vectors affected the protein expression of IFIH1 (*p* < .001), as shown in Figure S10.

### 3.11 IBR1 has no effect on M2 macrophage polarisation

To investigate whether IBR1 influences IFIH1, we used flow cytometry to assess the expression levels of CD206, a key marker indicative of M2 polarisation, in BMDMs from *WT* and *IBR1^−/−^* model mice. After stimulation with 20 ng/mL IL‐4 for 24 h with or without IBR1 overexpression, the cells were analysed.

The results indicated that the modulation of IBR1 expression does not affect the expression of the M2 macrophage surface marker CD206 (*p* > .05), as shown in Figure S11.

### 3.12 IBR1 involvement in LPS‐ and KP‐induced lung injury is mediated by IFIH1

**FIGURE 6:**
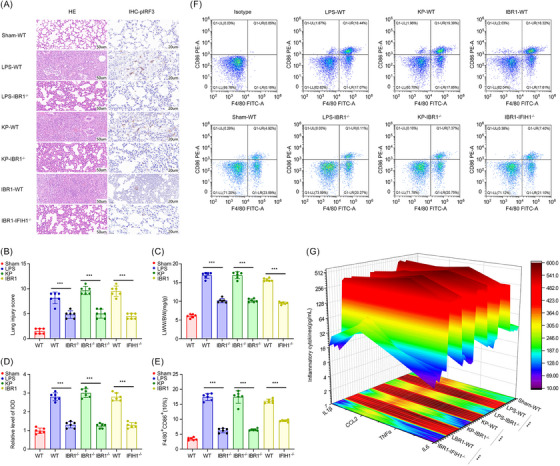
IFIH1‐binding RNA 1 (IBR1) involvement in lipopolysaccharide (LPS)‐ and *Klebsiella pneumoniae* (KP)‐induced lung injury mediated by IFIH1. (A) Histological evaluation by haematoxylin and eosin (H&E) staining was conducted to assess pulmonary injury induced by intratracheal instillation of LPS at a dose of 15 mg/kg, KP at a dose of 60 × 10^8^ CFU/kg, or IBR1 oligo (2 mg/kg) in *wild‐type (WT)*, *IBR1^flox/flox^Lyz2‐cre (IBR1^−/−^)* and *IFIH1^flox/flox^Lyz2‐cre (IFIH1^−/−^)* murine models, respectively. Immunohistochemical images were used to assess the protein expression of phosphorylated‐IRF3 (pIRF3) in murine pulmonary tissue. (B‒D) Lung tissue obtained from the designated experimental groups was assessed for its lung injury score and degree of lung oedema, which serve as histological indices of lung injury. Additionally, semiquantitative analysis of p‐IRF1 expression was conducted, as measured by integrated optical density (IOD). (E and F) Flow cytometric analysis was conducted to identify cells producing CD86 in the BALF collected 24 h after the administration of the LPS, KP or IBR1 oligo in the designated experimental groups. (G) Enzyme‐linked immunosorbent assay (ELISA) was conducted on BALF samples to assess the concentrations of proinflammatory cytokines, including interleukin‐1β (IL‐1β), chemokine (C‒C motif) ligand 2 (CCL2), tumour necrosis factor alpha (TNF‐α) and interleukin‐6 (IL‐6). Each group consisted of six mice. The presented data represent the mean ± standard deviation, and statistical significance was determined via appropriate statistical tests (^*^ *p *< .05, ^**^ *p *< .01, ^***^ *p *< .001).

Histopathological examination with H&E staining was performed to evaluate pulmonary damage caused by intratracheal administration of LPS, KP or IBR1 in murine models with different genotypes: *WT*, *IBR1^−/−^* and *IFIH1^−/−^* (Figure 6A). Following LPS or KP stimulation, WT mice exhibited more prominent features of congestion, alveolar collapse and alveolar wall thickening compared with those in *IBR1^−/−^* mice (Figure 6A). The lung injury score and degree of lung oedema were significantly reduced in the *IBR1^−/−^* mice (*p* < .001), as shown in Figure 6B,C.

Intratracheal injection of IBR1 could also induce inflammatory lung injury, but its effect was significantly attenuated in IFIH1 KO mice. This finding demonstrated that IBR1 serves as an intermediate mediator in LPS‐induced ARDS and that its actions are dependent on the IFIH1 pathway.

To indirectly evaluate the activation of IFIH1 in vivo, we examined pIRF3 levels through immunohistochemistry staining. Our results demonstrated that deletion of IBR1 significantly decreased the expression of pIRF3 in lung tissue from mice stimulated with either LPS or KP compared with that in lung tissue from *WT* mice stimulated with LPS or KP (*p* < .001). Furthermore, IBR1 was found to directly activate IFIH1 in vivo, as evidenced by the substantial reduction in pIRF3 expression upon IFIH1 KO (*p* < .001), as shown in Figure 6D.

To assess the impact of IBR1 on M1 polarisation of lung macrophages in ARDS, flow cytometry was conducted to detect the percentage of F4/80^+^CD86^+^ cells in BALF collected 24 h after LPS, KP or IBR1 administration in the different experimental groups. Moreover, we utilised ELISA to quantify the concentrations of proinflammatory cytokines, including IL‐1β, CCL2, TNF‐α and IL‐6, in the BALF.

The findings revealed that IBR1 deletion significantly reduced the number of M1‐polarised macrophages and the expression of proinflammatory cytokines in the BALF of the mice stimulated with LPS or KP compared with that in the BALF of the LPS‐stimulated *WT* mice (*p* < .001). Moreover, IBR1 was shown to directly induce the activation of macrophages towards the M1 phenotype in mouse lungs, thereby increasing the secretion of pulmonary inflammatory factors. Furthermore, KO of IFIH1 markedly diminished IBR1‐mediated pulmonary macrophage M1 polarisation in vivo (Figure 6E‒G).

These results suggested that IBR1 acts as an intermediary mediator in the polarisation of macrophages towards the M1 phenotype induced by LPS and KP, contingent upon the IFIH1 pathway.

### 3.13 Neutralisation of IBR1 as a therapeutic approach for ARDS

IBR1, an endogenous dsRNA found in the lungs during ARDS, has been shown to bind and activate IFIH1, inducing pulmonary inflammatory responses and leading to lung injury. Therefore, the exogenous administration of the IFIH1 mutant (IFIH1‐M), which retains only the helicase domain responsible for dsRNA binding while excluding other structural domains, may neutralise this dsRNA and alleviate pulmonary inflammation and lung injury. Macrophage‐derived EVs exhibit inflammatory properties. We utilised them as carriers for IFIH1‐M, and IFIH1‐M‐EVs were administered via tail vein injection at a dose of 1 × 10^10^ particles/mouse for the treatment of LPS‐induced ARDS.

**FIGURE 7:**
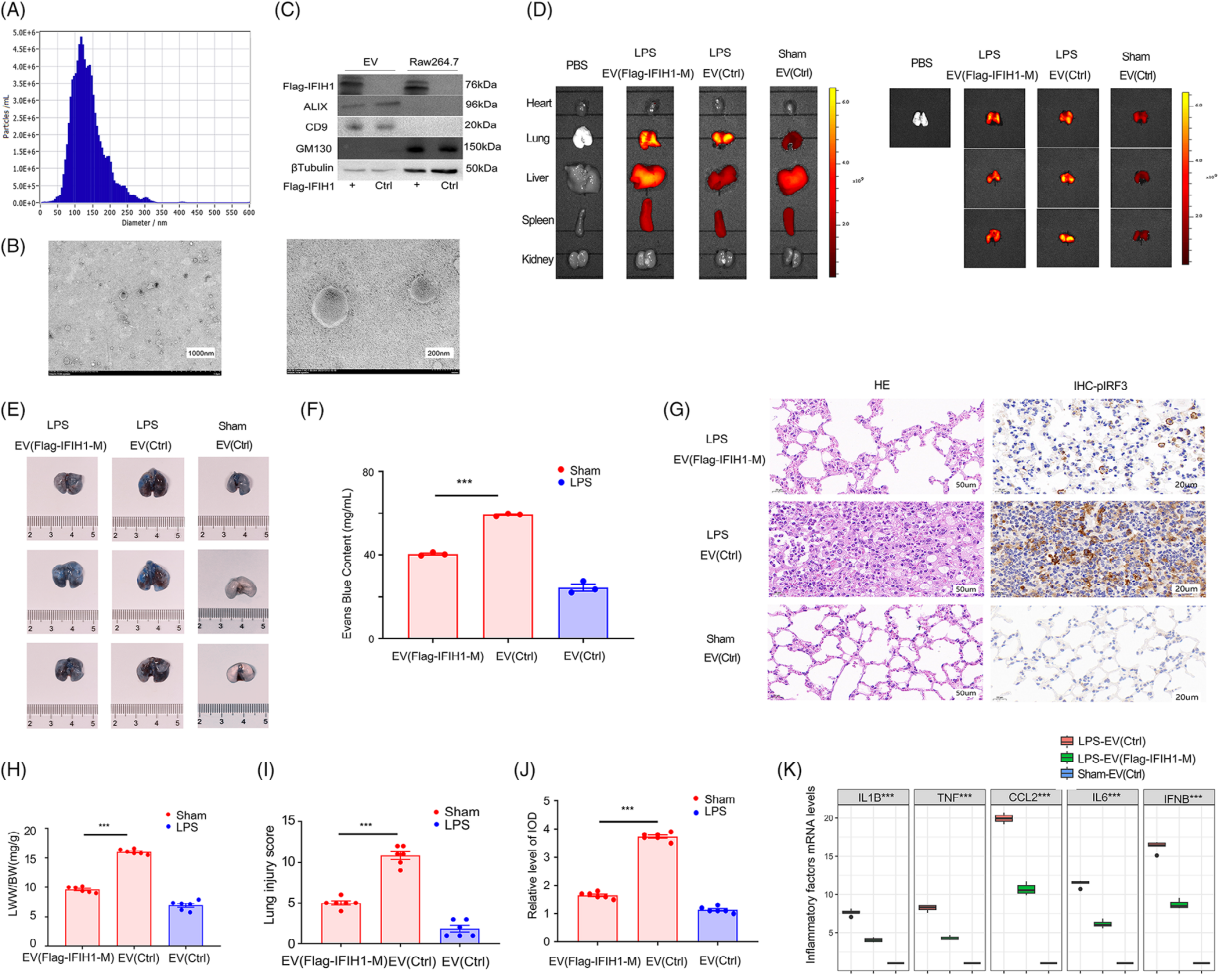
Neutralisation of IFIH1‐binding RNA 1 (IBR1) as a therapeutic approach for lipopolysaccharide (LPS)‐induced acute respiratory distress syndrome (ARDS). (A) The size distributions of exosomes (extracellular vesicles, EVs) obtained from the conditioned medium of RAW264.7 macrophages via nanoparticle tracking analysis (NTA) are presented. (B) Representative electron micrographs depicting EVs isolated from the conditioned medium of RAW264.7 macrophages. Scale bars: 200 and 1000 nm. (C) Western blot analysis was performed to evaluate the levels of EV markers (CD9 and ALIX), a negative marker (GM130), a macrophage marker (F4/80) and Flag in RAW264.7 macrophages and EVs with and without treatment with Flag‐IFIH1‐mutant (M) plasmids. (D) A representative fluorescence image illustrates the distribution of DiD‐labelled EVs in various organs across different groups 24 h after injection. (E and F) Effect of IFIH1‐M‐EV treatment on pulmonary vascular permeability according to the Evans blue assay. (G‒J) Histological evaluation via H&E staining was performed to assess pulmonary injury in subjects treated with IFIH1‐M‐EVs compared with untreated subjects in an LPS‐induced ARDS model. Representative immunohistochemical images depict the protein expression of pIRF3 in murine pulmonary tissue. Lung tissue from the designated experimental groups was analysed for its lung injury score and degree of lung oedema, which serve as histological indicators. Additionally, semiquantitative analysis of p‐IRF1 expression was conducted, as measured by the integrated optical density (IOD). (K) qPCR was performed to evaluate the levels of proinflammatory cytokines in pulmonary tissue homogenates.

To confirm the successful generation of IFIH1‐M‐EVs, we used NTA and assessed representative electron micrographs of EVs isolated from the conditioned medium of RAW264.7 macrophages (Figure 7A,B). Western blot analysis revealed enrichment of characteristic surface marker proteins of EVs (Alix, CD9 and CD63) in the EV samples, whereas the negative marker protein (GM130) was undetectable in the EVs (Figure 7C). Moreover, significant upregulation of the Flag‐IFIH1‐M protein was observed in EVs from the Flag‐IFIH1‐M protein‐transfected group (Figure 7C).

**FIGURE 8:**
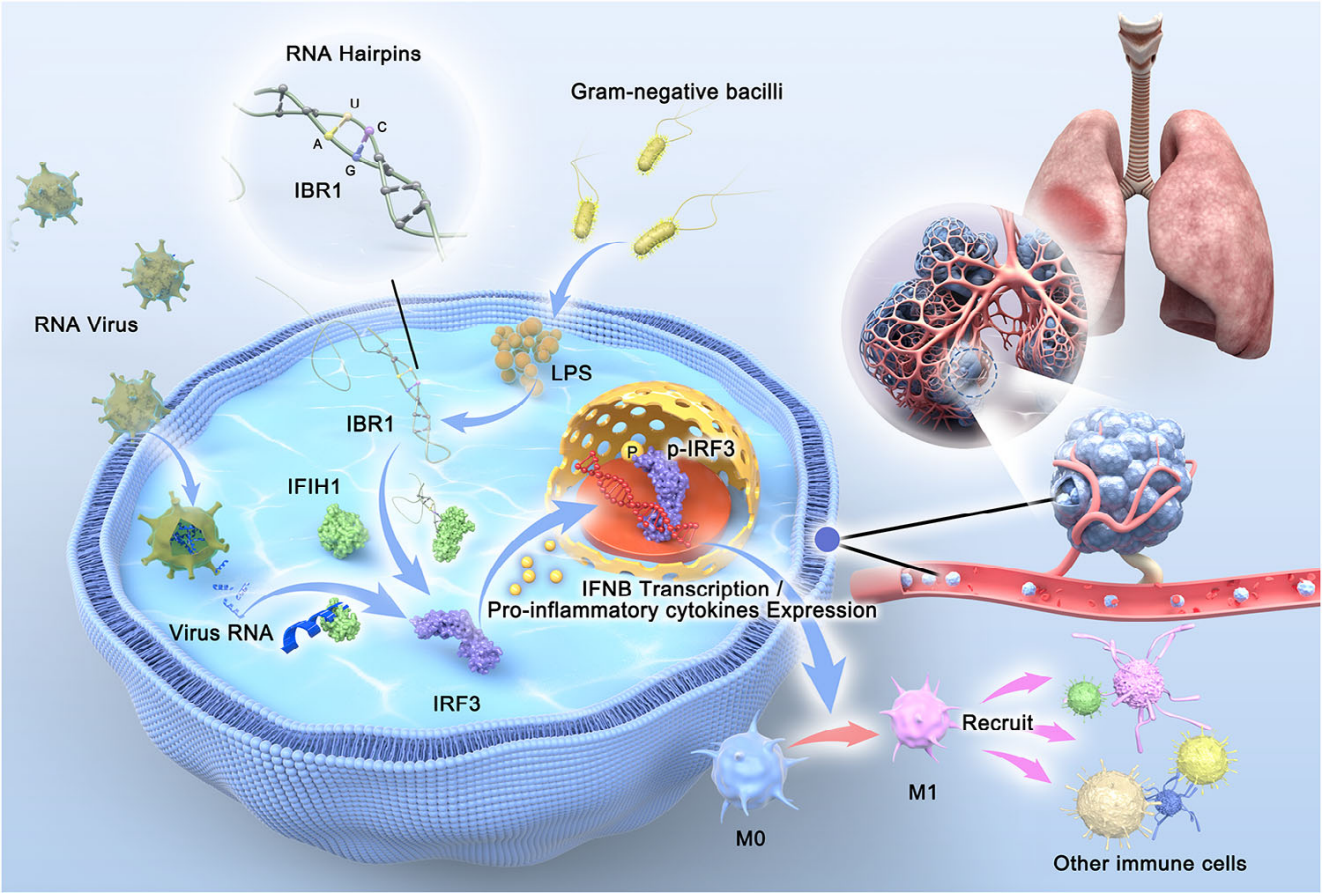
The graphical image summarises the main content of this article, illustrating the molecular mechanism by which IFIH1‐binding RNA 1 (IBR1) activates macrophages towards an inflammatory phenotype in acute respiratory distress syndrome (ARDS) through its binding and activation of IFIH1: the endogenous double‐stranded RNA IBR1 binds to the helicase domain of IFIH1 because of its unique double‐stranded structure. IBR1 plays a crucial role in macrophage polarisation and in the development of ARDS induced by Gram‐negative bacteria or lipopolysaccharide (LPS). Administration of IFIH1 variants has potential for eliminating pulmonary double‐stranded RNA (dsRNA) and reducing inflammatory lung injury in ARDS.

To assess the in vivo tissue distribution of IFIH1‐M‐EVs, mice were intravenously injected with DiD‐labelled EVs (1 × 10^10^ particles/mouse) or an equal volume of PBS. Ex vivo fluorescence imaging revealed robust accumulation of EVs in the lungs (Figure 7D), indicating effective recruitment to this organ.

To explore the therapeutic effects of IFIH1‐M‐EVs on LPS‐induced ARDS, we conducted experiments, including an Evans blue assay, pulmonary histopathological staining, lung wet‒dry weight ratio measurements, and analysis of inflammatory factors in the lungs. Compared with blank‐EVs, IFIH1‐M‐EVs significantly improved pulmonary vascular permeability (*p* < .001) (Figure 7E,F). Additionally, IFIH1‐M‐EV treatment significantly ameliorated LPS‐mediated lung injury, reduced the secretion of pulmonary inflammatory factors and concurrently attenuated IFIH1 activation (pIRF1 activation and IFNB transcription) (*p* < .001) (Figure 7G‒K).

To demonstrate that IFIH1‐M‐EVs can treat ARDS induced by dsRNA viruses, we constructed a dsRNA‐ARDS model with synthetic dsRNA (poly(I:C)) and administered IFIH1‐M‐EVs or Ctrl‐EVs via tail vein infusion to assess their therapeutic effects. Unlike the Ctrl‐EVs, the IFIH1‐M‐EVs markedly decreased inflammatory cytokine levels and alleviated lung injury in mice with dsRNA‐induced ARDS (Figure S12).

These findings suggested that IFIH1‐M‐EVs effectively neutralise both endogenous and exogenous dsRNA in the lungs, thereby alleviating the inflammatory lung damage associated with ARDS.

## 4 DISCUSSION

### 4.1 ARDS

ARDS is a common critical illness in clinical practice, with a mortality rate of 35%−61% for severe ARDS. This poses a significant challenge for clinicians in improving disease outcomes. Exploring its pathogenesis and developing new therapeutic targets are crucial for improving ARDS treatment success rates. Our findings provide evidence supporting the role of IBR1 as a novel molecule in mononuclear macrophages, contributing to LPS‐induced ARDS through macrophage M1 polarisation. ARDS is distinguished by excessive inflammation caused by macrophages and monocytes, leading to lung damage, commonly initiated by pneumonia.^14^, ^15^, ^16^ Gram‐negative bacilli are a common cause of pneumonia. Because LPS, a toxic element found in Gram‐negative bacteria, can trigger ARDS by inducing macrophage M1 polarisation, it has been commonly utilised to create animal models as well as in vitro models of ARDS with M1‐polarised cells.^17^, ^18^ Previous studies have primarily focused on the TLR4 pathway as the main molecular mechanism underlying LPS‐induced inflammation models.^19^, ^20^, ^21^ However, our investigation has revealed that IBR1‐mediated IFIH1 activation serves as an additional mechanism contributing to LPS‐triggered inflammation.^8^ Moreover, clinical samples have further confirmed the involvement of IBR1 in Gram‐negative bacilli‐induced ARDS. The findings improve our comprehension of ARDS pathogenesis, offering fresh perspectives on its core mechanisms.

Although we have confirmed that the LPS‒IBR1‒IFIH1 pathway can induce an inflammatory response, we have yet to verify whether the effects of LPS and IBR1 depend on LPS receptors, such as Toll‐like receptor 4. This will be further investigated in future studies.

### 4.2 Macrophage

Our current investigations have revealed that the binding of IBR1 to IFIH1 represents a novel molecular mechanism underlying LPS‐induced macrophage M1 polarisation. Macrophage polarisation is critical in many diseases, particularly in inflammatory conditions such as ARDS (Figure 8).^22^ Understanding the intricate molecular mechanisms that drive macrophage M1 polarisation is of great importance.^23^, ^24^, ^25^, ^26^, ^27^ In recent years, our research group has conducted a series of studies that have uncovered a novel pathway involving the binding of IBR1 to IFIH1, which contributes to macrophage M1 polarisation. These findings significantly enhance our understanding of the complex process of macrophage M1 polarisation.

Due to advancements in technologies such as single‐cell sequencing, more phenotypes of macrophages have been discovered. However, these sub‐phenotypes are still categorised under the broader M1/M2 phenotypes. Our project mainly elucidates the mechanism by which IBR1 influences the differentiation of macrophages towards the inflammatory phenotype. In future studies, our research team will continue to investigate the finer impacts of IBR1 on macrophage phenotypes.

### 4.3 IFIH1

IFIH1, a member of the RIG‐I‐like helicase family, serves as a conserved cytoplasmic sensor for viral RNA, specifically recognising dsRNA derived from a diverse array of viruses. It has been proposed that IFIH1 forms higher‐order structures upon recognition of viral dsRNA, thereby playing a role in antiviral signalling and the inflammatory pathway (Figure 8). IFIH1 consists of three distinct functional domains, with the helicase domain being identified as the site responsible for binding to dsRNA. Previous studies have provided evidence suggesting that IFIH1 can be activated by exogenous dsRNA, such as viral RNA and synthetic dsRNA mimics.^28^, ^29^, ^30^, ^31^ Our investigations have further revealed that IFIH1 can also be activated by endogenous dsRNA‒IBR1, induced by LPS in mononuclear macrophages. Through our in vivo and in vitro experiments, we have made significant advancements in understanding the mechanism of IFIH1 activation. Specifically, we have discovered that the 5′ terminal sequence of IBR1 contributes to the formation of double‐stranded structures that bind to the helicase domain of IFIH1, representing a novel mechanism of IFIH1 activation (Figure 8). These findings significantly enhance our understanding and knowledge of IFIH1, expanding our insights into the intricate interplay between dsRNA recognition and the subsequent immune response mediated by IFIH1.

RIG‐I and IFIH1 proteins function similarly by recognising viruses and binding dsRNA, then activating transcription factors such as IRF3/IRF7 to initiate antiviral and inflammatory pathways. Recent studies have found that endogenous RNA can also bind and activate IFIH1 and RIG‐I proteins, such as in our research (IFIH1‐binding RNA) and Jiang et al.’s research (RIG‐I‐binding RNA).^28^ Notably, we found that the IFIH1‐binding RNA‒IBR1 is dsRNA, while Jiang et al. did not further confirm whether the RIG‐I‐binding RNA is dsRNA or single‐stranded RNA (ssRNA). We speculate that the RIG‐I‐binding RNA discovered by Jiang et al. is also dsRNA, which aligns with the functional characteristics of RIG‐I and IFIH1 proteins (helicase domain recognising and binding dsRNA).

### 4.4 dsRNA

mRNA vaccines can produce transcriptional noise in the form of dsRNA during their manufacturing process. These dsRNA molecules, when introduced into the human body, can trigger inflammatory responses such as fever and headache. Therefore, eliminating dsRNA is a key aspect of RNA vaccine development. We found that endogenous dsRNA binds to IFIH1, leading to excessive inflammatory responses in the lungs. Clearing dsRNA from the body can effectively alleviate inflammatory lung injury.

Evidence shows that dsRNAs arise not only from viral infections but also from endogenous sources such as retroelements and mitochondrial DNA under various conditions. These endogenous dsRNAs trigger the same receptors as viral dsRNAs, signalling abnormal cellular processes and linking innate immune responses to diverse pathologies, from immune disorders to neurodegeneration.^32^, ^33^, ^34^, ^35^, ^36^ In a newer hypothesis, it has been suggested that the activation of IFIH1 is primarily triggered by inverted sequence repeats positioned adjacently, resulting in the creation of extensive hairpin structures.^37^, ^38^ This hairpin formation mechanism is likely related to the re‐binding of RNA polymerase to the 5′ end of the transcript after transcription is completed. Our study provides empirical validation for this hypothesis. Specifically, we have identified multiple complementary bases (TAAGGGCTGCGCTGGAAC‐GTTCCAGCGCAGCCCTTA) at the 5′ end of the IBR1 sequence, which are prone to inward folding and subsequently form double‐stranded structures known as RNA hairpins (Figure 8).

IBR1 has the sequence basis to form double‐stranded structures (complementary base sequences), while IBR2 does not. Dot plot experiments further confirmed that IBR1 is dsRNA, whereas IBR2 is ssRNA. dsRNA can bind to the helicase domain of IFIH1, leading to IFIH1 activation. In contrast, ssRNA lacks the structural basis to bind to the helicase domain of IFIH1, rendering its binding to IFIH1 biologically insignificant and unable to activate IFIH1. The discovery of IBR1 enriches the theoretical framework of endogenous dsRNA and its consequence in immunity.

### 4.5 Treatment

IBR1, a type of dsRNA, serves as a crucial mediator of inflammatory pathological damage in ARDS. Neutralising IBR1 holds the potential to disrupt dsRNA‐mediated inflammatory responses. The IFIH1 protein is made up of three domains the helicase domain, which binds to and unwinds dsRNA; the CARD domain, which interacts with the downstream protein MAVS, triggering pathways like IRF3 and leading to inflammatory responses. By engineering a mutant variant of IFIH1 (IFIH1‐M), we retained only the helicase domain responsible for dsRNA binding while removing other domains involved in activating downstream inflammatory pathways. This IFIH1‐M variant may act as a ‘dsRNA scavenger’, potentially alleviating dsRNA‐induced inflammatory lung injury. Previous studies have demonstrated the inflammatory targeting capability of macrophage‐derived exosomes. Therefore, we utilised macrophage‐derived exosomes as carriers for IFIH1‐M. The results also confirm that IFIH1‐M‐EVs significantly mitigate inflammatory lung injury induced by LPS.

The IFIH1 we administered is a mutant protein that retains only the function of binding dsRNA (the helicase domain), while the function of activating the downstream inflammatory pathway (the CARD domain) has been delete. Therefore, even in patients with dsRNA viral pneumonia, IFIH1‐M‐EVs can still effectively clear exogenous dsRNA viruses, reducing viral titres while alleviating dsRNA virus‐mediated inflammatory responses. To confirm this, we have supplemented the experiment. We used dsRNA‒poly(I:C) to establish a dsRNA‒ARDS mouse model and administered IFIH1‐M‐EVs via the tail vein. The results indicate that IFIH1‐M‐EVs can effectively mitigate inflammation‐mediated lung injury caused by both dsRNA and LPS.

## 5 CONCLUSION

Our research provides strong evidence supporting the role of IBR1 in Gram‐negative bacteria‐induced ARDS by promoting macrophage M1 polarisation. We have elucidated a novel mechanism wherein IBR1 interacts with IFIH1, shedding light on the intricate process of macrophage polarisation. Furthermore, we have enhanced our understanding of the interplay between endogenous dsRNA and the IFIH1‐mediated immune response. Exogenous infusion of IFIH1 variants shows potential for clearing pulmonary dsRNA and mitigating inflammatory lung injury in ARDS patients. These findings contribute to our knowledge of ARDS pathogenesis and offer new insights into potential therapeutic targets for this debilitating condition. The study findings are summarised in Figure 8.

## AUTHOR CONTRIBUTIONS

Shi Zhang, Jianfeng Xie, and Haibo Qiu conceptualised the study. Shi Zhang conducted the experiments, analysed the data and drafted the manuscript. All authors contributed to the discussion of the study.

## CONFLICT OF INTEREST STATEMENT

The authors affirm that they do not have any competing financial interests or personal relationships that may have impacted the findings presented in this paper.

## ETHICS STATEMENT

The current study was conducted in accordance with the revised Helsinki Declaration. Biological specimens utilised in this study were obtained from the ARDS biobank at the Affiliated Zhongda Hospital of Southeast University and the Central Hospital Affiliated to Shandong First Medical University. The establishment of the specimen bank was approved and overseen by the Institutional Ethics Committee of Zhongda Hospital and the Central Hospital Affiliated to Shandong First Medical University. Ethical documents related to this study can be accessed in the attached Supporting Information. Animal research conducted in this study was evaluated and authorised by the Animal Care and Use Committee of the Central Hospital Affiliated to Shandong First Medical University. Further details regarding this authorisation can be found in the attached Supporting Information.

## Supporting information



Supporting Information

## Data Availability

The RNA‐seq and RIP‐seq datasets produced in this research have been stored in the GEO database with the accession codes GSE244502, GSE247176, GSE247177 and GSE245440. All other data associated with this study can be found in the Article or Supporting Information. For additional information or data requests, please contact the corresponding author.
