# Supplementary material for: IBR1, a novel endogenous IFIH1‐binding dsRNA, governs IFIH1 activation and M1 macrophage polarisation in ARDS
Source: Clin Transl Med. 2024 Sep 23;14(9):e70027. doi: 10.1002/ctm2.70027 (PMC11420289; doi:10.1002/ctm2.70027)
Supplement: Supplementary file 1 — Supporting Information [file CTM2-14-e70027-s001.docx]

Table S1. The information of IBR1

| **IBR1 in Mouse Genetic Contexts** |
| --- |
| chr13:108494843-108498957 |
| TGTATTTTCTCCTTATAATCACTCCCTCCCGGGGCATGTAATTAATTTCCTGATGGATTACTCACACGGTCTTGAAGATGCAATGTCAGCTATTTAGGCTGTGCTAGGACTCAATTACAACAACATATGCATTAAGACAGGAACTGGCAAGCCGGGACTACAGCAACTACAGGGCTGGTGCTTCCCAACCGCTGGGCTATAATCTATGAAACTGAGCCGGGATCCAGCCAATCAGTTAGCTAGCTCCTCATAACAGGTCTAACTGGCTCTGGACAGCTAAGGGCTGCGCTGGAACGTTCCAGCGCAGCCCTTA |
|  |
| **IBR1 in Human Genetic Contexts** |
| chr5:60487712-60491518 |
| AAACAGCCTCCTTCAAAATGCAGCTTGACTGTGATTCATGGCACAAAGCT  GGAATAACCCTGCCAGTTCTTAGAAGCTTTAAGATTCATCGTCTTGAACT  TGATACACATAGTATAGCACCTTAAAAACGAATCATCTGCTAGTTCAGTA  TTTTCTACTTACAATCACTCCCTCCCAGGGCATGTAATTAATTTACTGAG  CTTAAAAAAAAAATATCAGGATTACTCACACAGTCTTGAAGATGCAATGT  CAGCTATTTAGGACAGAAACATCCAAGGCCGTGTCAGAACTCAATTACGA  CTACATATGCATTAAGGCAGGAACTGGCAGGCCTCAGGGTACGCCAACTA  TAGGACTCGTGCTTCTCGTACGCTGGGCTATAATCTATGAAACTGAGCTC  CAGAGCCAGCCAATCACTTAGCTCCTCATAACAAGTCTAACTGGCTCTGG  AAAGCTGAAAGGGCTGCACTGGAACAACACAGATGAGATATTCTACACAT  TAATCTACTTATCTGGAATCACTTTGCCTCTAAAGGCCAGAGAAAAATCA  CAGCTTCCTTGTCGGAGGGGAAAAGGACAGGTGATCTGGGGAAAACGCAG  CTACACCTGGAGCAAGGTCTCTTCCCGGCTTGGCAATCTCAGCTGTGCCG  GCGCTACGGGACCCGAGCCGTCCCAGAAACCAAAGGGCAGGCACGGCAGC  AAACGCCTGAGTGCTGCTGCCTTCGGTGACTATATGAGAATGGAAACTTC  TAAGGAAGCCAGGTTGTTAGAATTGTTACCCCCTTTACTCAGAGATAACA  TAGATTATCCAGGCTGAGATGGAAAACAAGCCCTTTATTGAATTTTCAAC  ACAGACTCCCTGCTTCTCATCTCCTTAATAAAATTTCATTAAAATCCCCT  TGAACTCCCATGTTCAAATCTCCATTTGTTGACAGACAAAGCCAACAATACTC  TAAACTGAGGCCTGCAAGTCATTTCATTTGTATTTTTGTCCAGAAAT  TTCCCATAGGAAGACTTCACCTCCTACAACTCCGAAGAAAACCCTTACTG  TCCAAGACCGTCACCAGCAACCATCCGCAGTCATTCAAGTGGAAGCTTTC  ACAGCTTTTGTACATTCTCTGTGTCAATATACAACTGAGTTACAGACTGT  CCCCTGGCTCCCTGACCCTTACAAACACTAAAAGTTTTGTTTGACTCAAC  TTCAAGCTGCTCATCTGTTAGTAAGTGATGTTCACTCCAGAACACATTCA  TGATGAGAACTTTCTAAAAGACCAGCACTGCTCTTCCCCTCCTATAATCA  TAATAATCATGATAACCTGAAACATGTTACTGGGACTCGACATTTTTCTG  GGGATTGAAATCTTTAGTCCTTGGAGCTGTCACATAGCAGGGGCAACCTC  ACACTGAAACAAAGGAAGTGATGTCCCATTATTATCCACCCTGAGCCACC  ATAATATGCTGTTTACATTTATTTTCTTCAGCCTGTGCAAAACAAAGCAA  TGGAAAAGGAAACTAAAAAATATACATACTAGTACCATTATCTTCTTTTG  CCTAAAATTACTAATGCACCACGTCAGTCTGCTTCCTTCAGGCATCATTC  TCAATTCATCAGGACTTGTATTAGCAGGTTCTGGCTAGAGAGACTATCTC  CTGTCATCACGATCAATTAATGTTTTCTGGTGATCACATCAGGCCCTATC  TAAGAAGCTCATGGTATACAAGGGTCACCCAAATAGCTGAGTGCAGTCCT  TGCTCATATTTCCTTCATCTTAACCCCGCAAACAAGAATTAAGATGATCC  CAATAAAAGAAAAATTGCTCAGGAAACTGAACCTTTTTCTGAACCAAGCA  CTGTCAGCAAATCTCAGGTATTAGAGCAACTATGGTTGATTGAAAAGTGT  CTCAAAATCTGGGCCAAGAATGATTGCTAGGTCCATAAGCTAATTTGTCT  GGCCTTGCCATTTACGTAAGCCAAAGAAAGTCACTCATGAGTAAACTATA  GAAAACGTTCAGACCCATCCTGTTAGTATGTCAAATCAACTAAGACTGGC  AGGGTATTAACTCCATTCCAGGTGACATGGATAAAGAGCCCCATTATTTT  CACAGTGCCAGCCTCTACCTAAGGAAACCCTAGACCTTGGAACCAGTTTC  CTGGTAGGGAACTGCTGACAGTTTCAATGCTGACAGTTGGAGCCAATGCC  TCATAGTGTAAACTGAAAGAAAAATAGTTGCTTTTTAAAATGTCAGCAAG  AAGGCCTGCCTCATCTTAACAAAGCAAAAAAAAATGCTTTAATTCAAATT  AAAAATCATGATACTAGAGATGGGAGTCTGTGGTCACTATAAAGATAAAG  TTCATGTTCTTGGTTCAGATAACAAAACACTTTATCTGGATAAATGGACC  TCAAAATTTGCTCTTGGCACATGGGGGAAATtttttgttgtattttgttg  ttgttgttgttcttgttgttattgttTAAATTAATCTTCCTAAGGCCATG  AATATGAAATCATATTAATAATGCATGTGACAAAATTCTAAAATATATTACTGTTTTAAGATTTATA |

Table S2. Baseline Characteristics of All Patients

| Characteristic | Total (140) | Respiratory viruses (33) | Gram positive bacteria (33) | Gram negative bacteria(42) | Atypical bacteria (32) |
| --- | --- | --- | --- | --- | --- |
| Age,  mean (SD), y | 68.11±4.95 | 66.27±7.07 | 68.91±5.66 | 69.78±21.92 | 66.97±16.35 |
| Sex, No. (%) | | | | | |
| Male | 89(63.60) | 23(69.70) | 19(57.58) | 26(61.90) | 21(65.63) |
| Severity | | | | | |
| APACHE II, mean (SD) | 19.94±4.24 | 18.18±0.71 | 21.27±4.24 | 21.50±6.92 | 18.31±5.26 |
| SOFA, mean (SD) | 9.67±3.49 | 9.18±3.90 | 9.96±3.72 | 10.26±3.02 | 9.16±3.37 |

APACHE: Acute Physiology and Chronic Health Evaluation; SOFA: Sequential organ failure score; SD: Standard Deviation.

Table S3. Primers of overexpression vector

| IBR1 | F: TACTCTAGAGCTAGCGAATTCTGTATTTTCTCCTTATAATCAC  R:TAGAAGGCACAGTCGGCGGCCGCCAAGGTCGCGTCGGGAATGTTC |
| --- | --- |
| IFIH1-WT | F: aactttaagaaggagatataccatggATGTCGATTGTCTGTTCTGC  R:TCGTCATCGTCTTTGTAGTCgccgctgctATCTTCATCACTATACAAGC |
| Flag | tggtgctcgagtgcggccgcaagcttTCACTTGTCGTCATCGTCTTTGTAGTC |
| IFIH1-CARD1 | F: aactttaagaaggagatataccatggATGGCAGAGGACAGCTTCAGG  R: TGGAGACACTCGTCATGGGCGGGTTTGACATAGCGCGCGGC |
| IFIH1-CARD2 | F: AGCCGCGCGCTATGTCAAACCCGCCCATGACGAGTGTCTCCACT  R:GTCGTCATCGTCTTTGTAGTCgccgctgctATTTCCAGTTTGGCGCAGAAC |
| IFIH1-helicase | F: ttaactttaagaaggagatataccatggATGGCCCAACCAGCTCTAGATG  R:TCGTCATCGTCTTTGTAGTCgccgctgctCTTATGTGCATACTCCTCTGG |
| IFIH1-CTR | F: taactttaagaaggagatataccatggATGGCAAAGCAATACAACGACAA  R:TGTCGTCATCGTCTTTGTAGTCgccgctgctCAAGCAGTATTCTGAGTAGTC |

Table S4. T7 RNA synthesis

| IBR1 | TGTATTTTCTCCTTATAATCACTCCCTCCCGGGGCATGTAATTAATTTCCTGATGGATTACTCACACGGTCTTGAAGATGCAATGTCAGCTATTTAGGCTGTGCTAGGACTCAATTACAACAACATATGCATTAAGACAGGAACTGGCAAGCCGGGACTACAGCAACTACAGGGCTGGTGCTTCCCAACCGCTGGGCTATAATCTATGAAACTGAGCCGGGATCCAGCCAATCAGTTAGCTAGCTCCTCATAACAGGTCTAACTGGCTCTGGACAGCTAAGGGCTGCGCTGGAACGTTCCAGCGCAGCCCTTA  **3’5’-C6 Biotin** |
| --- | --- |
| IBR2 | GTGAAAATGAGGTAGGGAAATTGTTATTTATCACAGAAATCCCAGAATTAATACTAGAAGACCCCAGTGAAGCCAAAGAGAACCTCATCCTACAAGAAACATCTGTGGTAGAGTCACTGGCTACGGATGGGAGCCCAGGACTGAAATCAGTGCTGTCTACAGGCCGAAATCCAAGCAACAGCTGTGACTCAGGAGAGAAACCAGTGGTCACCTTTAAAGAGAACATTAAGCCACGAGAAGTGAACCAAGGAAGAAGCTTTCCTCCCAAAGAGGTAAAATCCCAGACAGAACTAAGAAAGACTCCAGTGTCTGAAGCCAGGAAAACGCCTGTCACTCAAACTCCAAGTCAAACGAGTAATTCTCAGTTCATCCCCATCCATCACCCTGGAGCCTTCCCTCCTCTTCCCAGCCGACCAGGTTACACCTTCCCAGCTGGTGTTTCTGTCCCAGGAACCTTTCTTCAGTCTACAGCTCACTCTCCAGCAGGAAACCAGGTGCAAGCTGGGAAACAGTCCCACATTCCTTACAGCCAGCAACGGCCCTCTGGACCAGGGCCAATGAACCAGGGACCTCAACAGTCACAGCCACCTTCCCAGCCACCCCTTACATCTTTACCAGCTCAGCCAACAGCACAGTCTACAAGCCAATTGCAGGTTCAAGCTCTAGCTCAGC  **3’5’-C6 Biotin** |
| IBR1-M1 | TAAGGGCTGCGCTGGAACGTTCCAGCGCAGCCCTTA |
| IBR1-M2 | TGTATTTTCTCCTTATAATCACTCCCTCCCGGGGCATGTAATTAATTTCCTGATGGATTACTCACACGGTCTTGAAGATGCAATGTCAGCTATTTAGGCTGTGCTAGGACTCAATTACAACAACATATGCATTAAGACAGGAACTGGCAAGCCGGGACTACAGCAACTACAGGGCTGGTGCTTCCCAACCGCTGGGCTATAATCTATGAAACTGAGCCGGGATCCAGCCAATCAGTTAGCTAGCTCCTCATAACAGGTCTAACTGGCTCTGGACAGCTAAGGGCTGCGCTGGAAC |
| IBR1-M3 | GTTCCAGCGCAGCCCTTA |

Table S5. Antibodies

| **Protein** | **Information** |
| --- | --- |
| **Western blot/ Immunohistochemistry** | |
| iNOS | Abcam, rabbit mAb, #ab178945 |
| βTubulin | Abcam, rabbit mAb, #ab108342 |
| GAPDH | Abcam, rabbit mAb, #ab181602 |
| IRF3 | Abcam, rabbit mAb, #ab68481 |
| pIRF3 | Cell Signaling Technology, rabbit mAb, 29047 |
| IFIH1 | Cell Signaling Technology, rabbit mAb, #5321 |
| F4/80 | Abcam, rabbit mAb, #ab300421 |
| Flag | Abcam, rabbit mAb, #ab205606 |
| ALIX | Abcam, rabbit mAb, #ab275377 |
| CD9 | Abcam, rabbit mAb, #ab307085 |
| GM130 | Cell Signaling Technology, rabbit mAb, #70767 |
| **Flow Cytometry** | |
| CD80 | FITC anti-mouse CD80, biolegend, Armenian Hamster, mAb, #104705 |
| CD86 | PE anti-mouse CD86, biolegend, Rat, mAb, #159203  APC anti-mouse CD86, biolegend, Rat, mAb, #105012 |
| F4/80 | FITC anti-mouse F4/80, biolegend, Rat, mAb, #123107  PE anti-mouse F4/80, biolegend, Rat,mAb, #111704 |
| CD206 | FITC anti-mouse CD206, biolegend, Rat,Recombinant, #162508 |
| **Dot plots** | |
| J2 | Abcam, rabbit mAb, # ab288755 |
| **RNA fluorescence in situ hybridization** | |
| IFIH1 | Invitrogen, rabbit mAb # 33H12L34 |
| CD14 | Proteintech, rabbit pAb # 17000-1-AP |
| **Immunoblotting and RNA immunoprecipitation** | |
| IFIH1 | Cell Signaling Technology, rabbit mAb #5321 |
| Flag | Abcam, rabbit mAb, #ab205606 |
| IgG | Santa Cruz Biotechnology, sc-2025 |

Table S6. PCR primers

| IBR1-mouse | F: CTCACACGGTCTTGAAGATGC  R: CCTTAGCTGTCCAGAGCCAG |
| --- | --- |
| IBR1-human | F: ACAGACAAAGCCAACAATACTC  R: GACAGTCTGTAACTCAGTTGT |
| GAPDH | F: AGCTTCGGCACATATTTCATCTG  R: CGTTCACTCCCATGACAAACA |
| IFNB mRNA | F: AGCTCCAAGAAAGGACGAACA  R: GCCCTGTAGGTGAGGTTGAT |
| IBR1^-/-^ Genotype | F: CTGAGTCCAGGGAGGTTTGTGT  R: ATAGAGGGAGATAGTAAAACTCTGCT |
| IFIH1^-/-^ Genotype | F: TCCAAGGCAGTGCATTAGTTG  R: TCATGCTTTGTTTGGCGGTG |
| IL1B | F: GCCACCTTTTGACAGTGATG  R: CGTCACACACCAGCAGGTTA |
| TNF | F: GATCGGTCCCCAAAGGGATG  R: TGAGGGTCTGGGCCATAGAA |
| CCL2 | F: ACAAGAGGATCACCAGCAGC  R: GGACCCATTCCTTCTTGGGG |
| IL6 | F: GCCTTCTTGGGACTGATGCT  R: GACAGGTCTGTTGGGAGTGG |


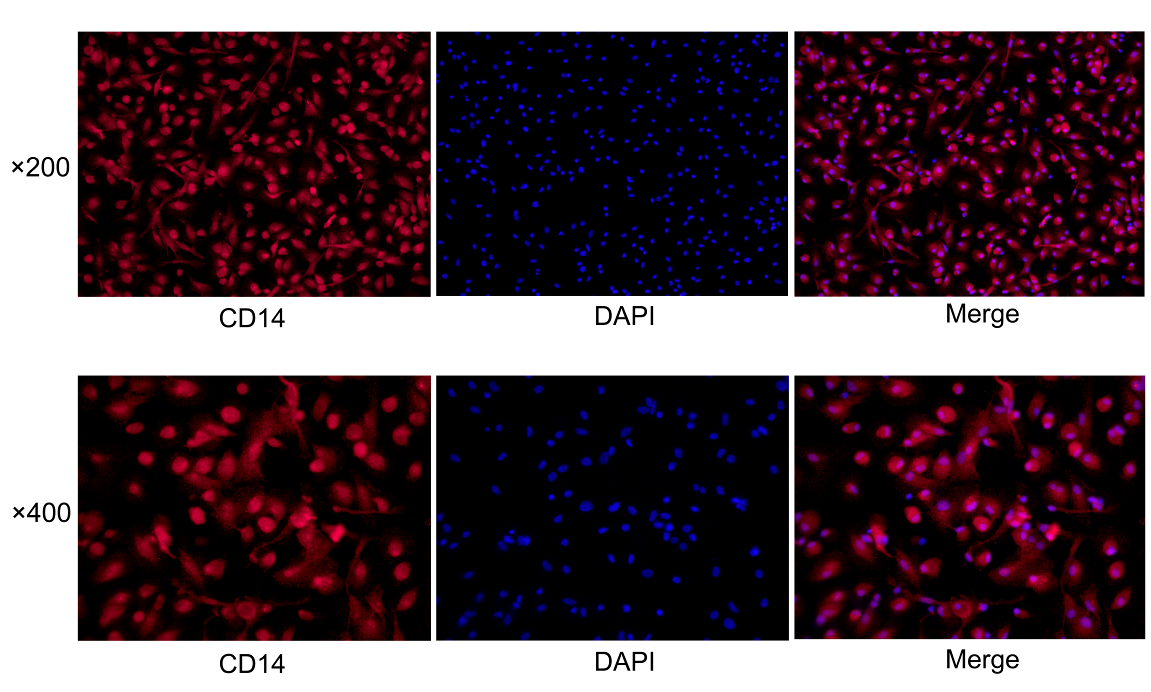


**Figure S1. Identification of isolated human CD14^+^ monocytes through immunofluorescence**


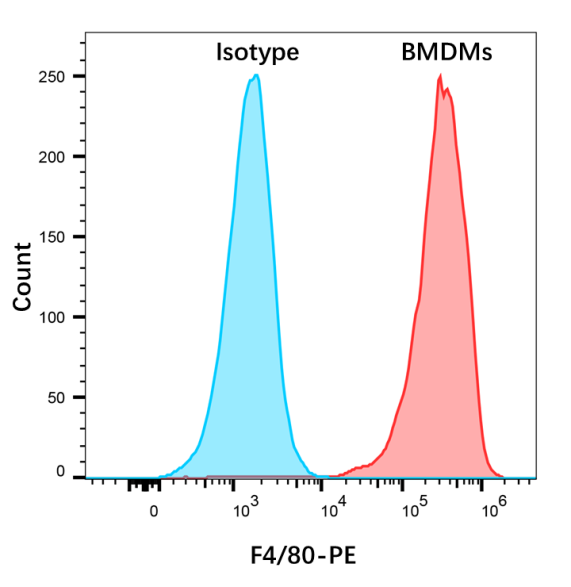


**Figure S2. Identification of isolated BMDMs through flow cytometry**

**
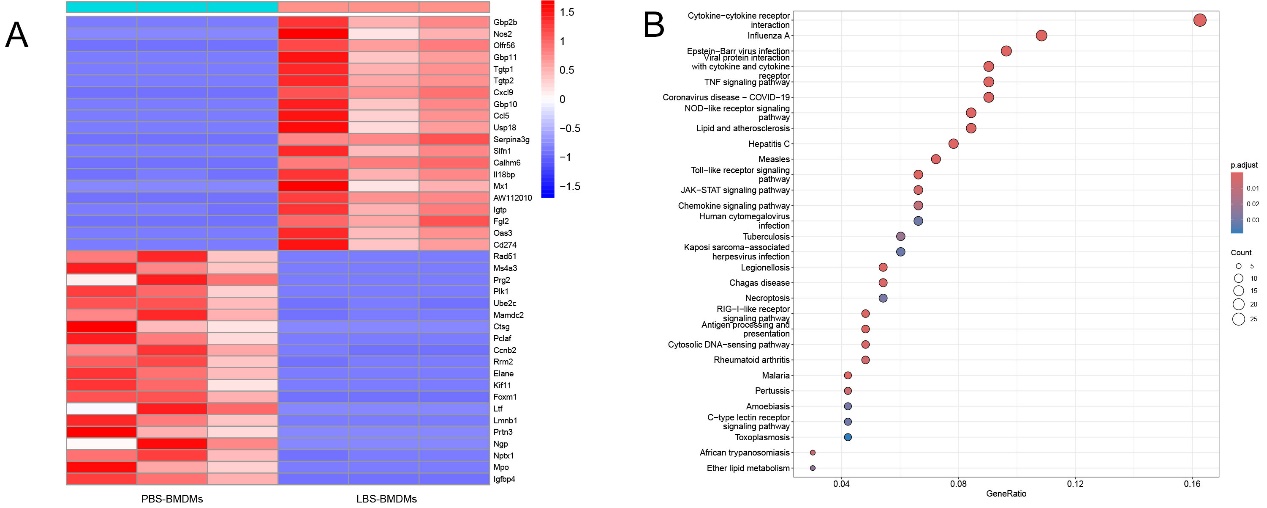
**

**Figure S3. The Top 20 Differentially Expressed Genes and KEGG Analysis Between LPS-BMDMs and PBS-BMDMs**


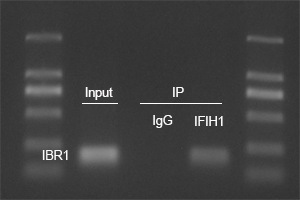


**Figure S4. IBR1 binds to IFIH1 in T cells**

RIP-PCR detection for the enrichment of IFIH1 on IBR1 in CD4^+^ T cells. IgG as the negative control.


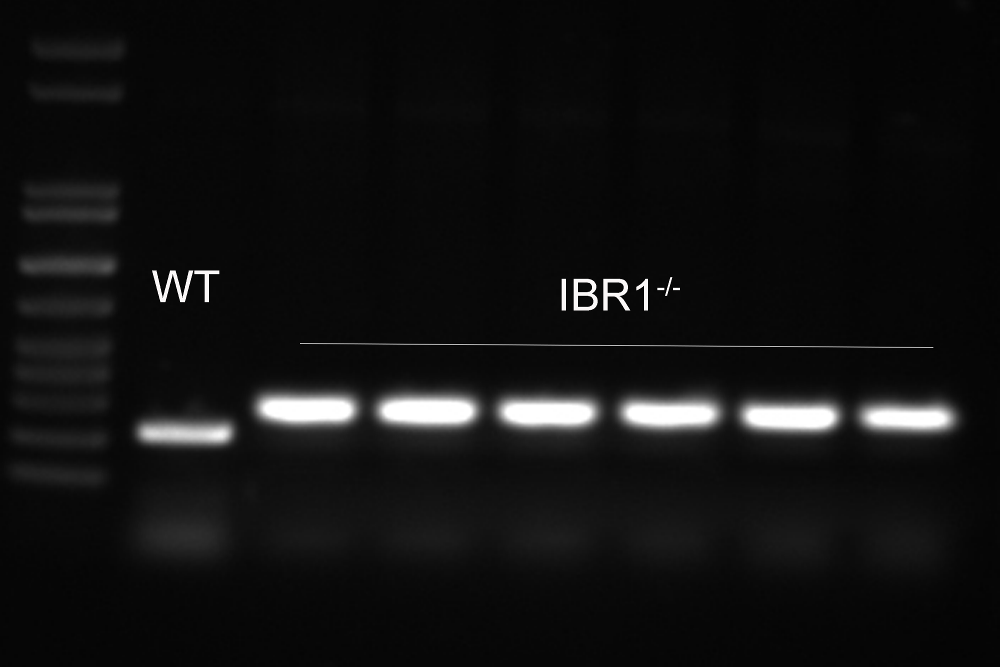


**Figure S5. Genotype of *IBR1^flox/flox^Lyz2-cre* mice**

The DNA from mouse tail tissue was extracted and amplified using PCR. The flow primers were designed and amplified using PCR as well. Agarose gel electrophoresis imaging was conducted to identify the flow genotype. The genotypes are as follows:

1: *Wild Type (WT)*

2-7: *IBR1^flox/flox^Lyz2-cre (IBR1^-/-^)*homozygous mouse.


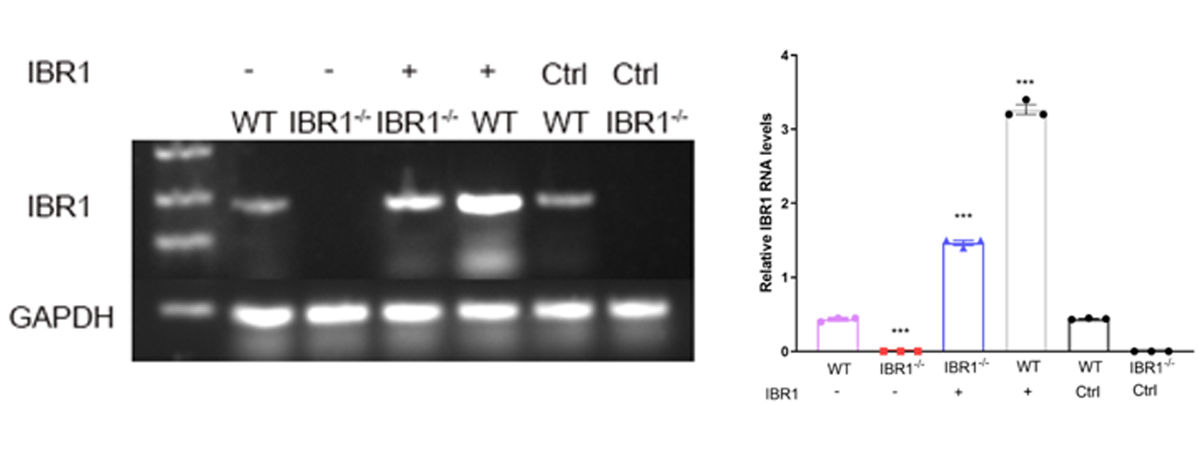


**Figure S6. The knockout, rescue, and overexpression efficiency of IBR1 in BMDMs.**

(1)Knockout Efficiency:PCR was performed using specific primers to detect the presence or absence of the IBR1 gene in *IBR1^flox/flox^Lyz2-cre* BMDMs. The absence of the IBR1 gene indicates successful knockout of IBR1 in the BMDMs.

(2)Rescue Efficiency:For rescue experiments, *IBR1^flox/flox^Lyz2-cre* BMDMs were transfected with a plasmid containing the IBR1 gene.

(3)Overexpression Efficiency:*IBR1^flox/flox^Lyz2-cre* BMDMs were transfected with a plasmid containing an overexpression construct of the IBR1 gene.

PCR identification allows for the assessment of the knockout, rescue, and overexpression efficiency of IBR1 in BMDMs.


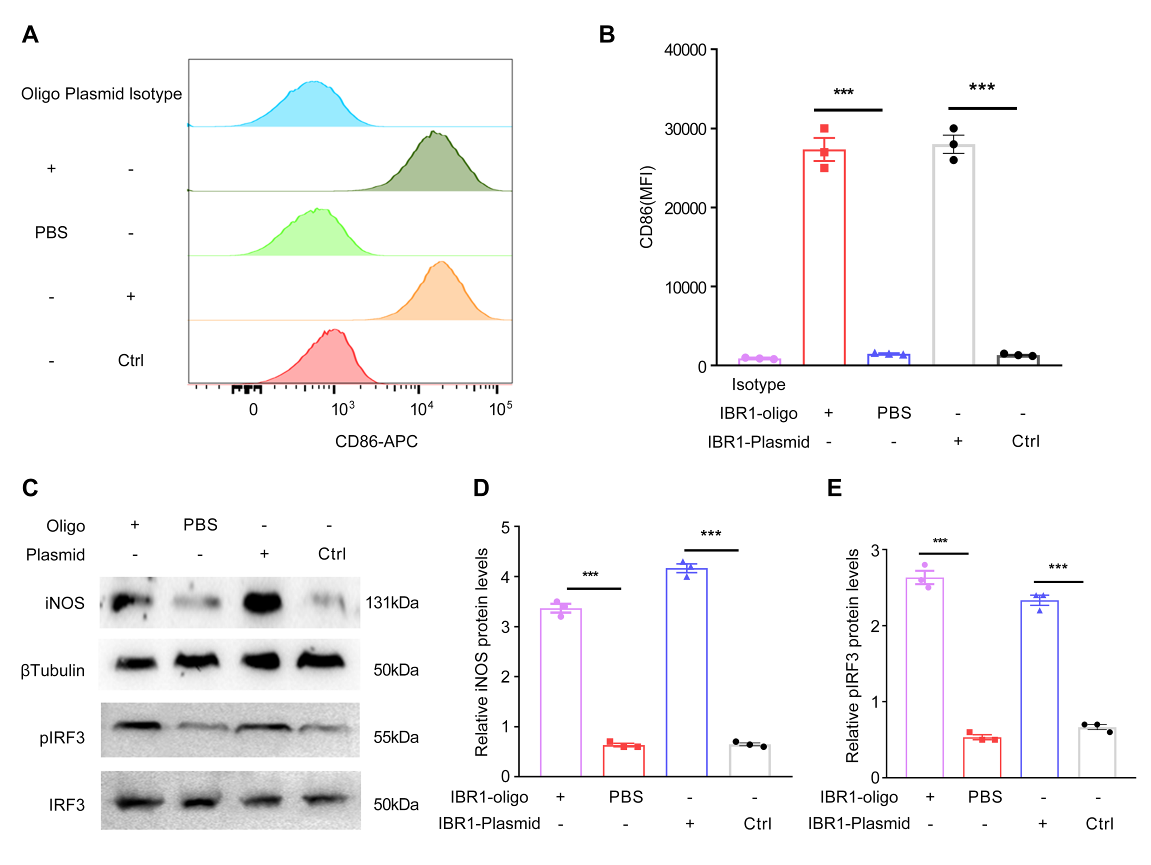


**Figure S7.IBR1 directly induces macrophage M1 polarization and IFIH1 activation**

(A and B) Flow cytometric analysis was conducted to measure the production of CD86 by Bone Marrow-Derived Macrophages (BMDMs) treated with IBR1 oligo (10ng/mL), plasmid IBR1 expression vectors (1ng/mL), and blank controls.

(C and D) Western blot analysis and quantitative analysis were performed to determine the protein levels of iNOS and p-IRF3 in the BMDMs from the specified experimental groups.

The presented data represents the mean ± standard deviation, and statistical significance was determined using appropriate statistical tests (**P*< 0.05, ***P*< 0.01, ****P* < 0.001).


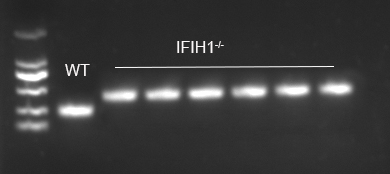


**Figure S8. Genotype of *IFIH1^flox/flox^Lyz2-cre* mice**

The DNA from mouse tail tissue was extracted and amplified using PCR. The flow primers were designed and amplified using PCR as well. Agarose gel electrophoresis imaging was conducted to identify the flow genotype. The genotypes are as follows:

1: *Wild Type (WT)*

2-7: *IFIH1^flox/flox^Lyz2-cre (IFIH1^-/-^)* homozygous mouse.


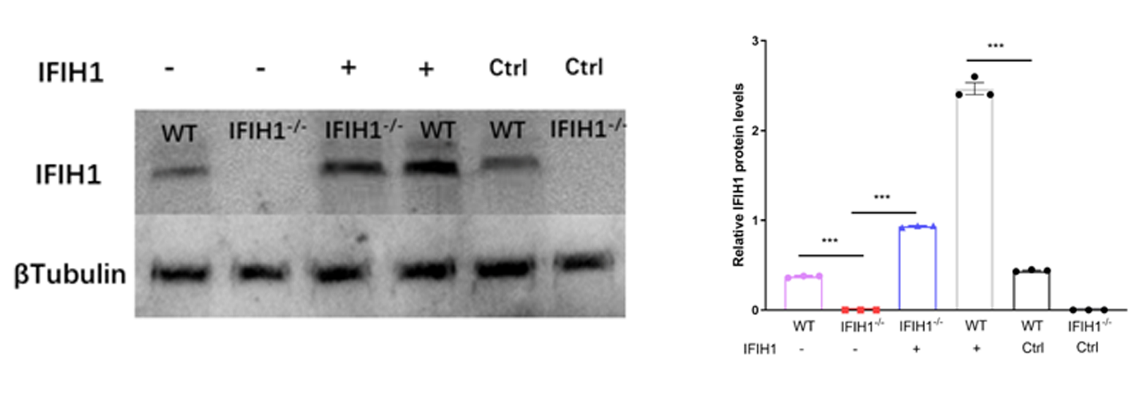


**Figure S9. The knockout, rescue, and overexpression efficiency of IFIH1 in BMDMs.**

Western blots identification allows for the assessment of the knockout, rescue, and overexpression efficiency of IBR1 in BMDMs from *IFIH1^flox/flox^Lyz2-cre* and *Wild Type*mice.


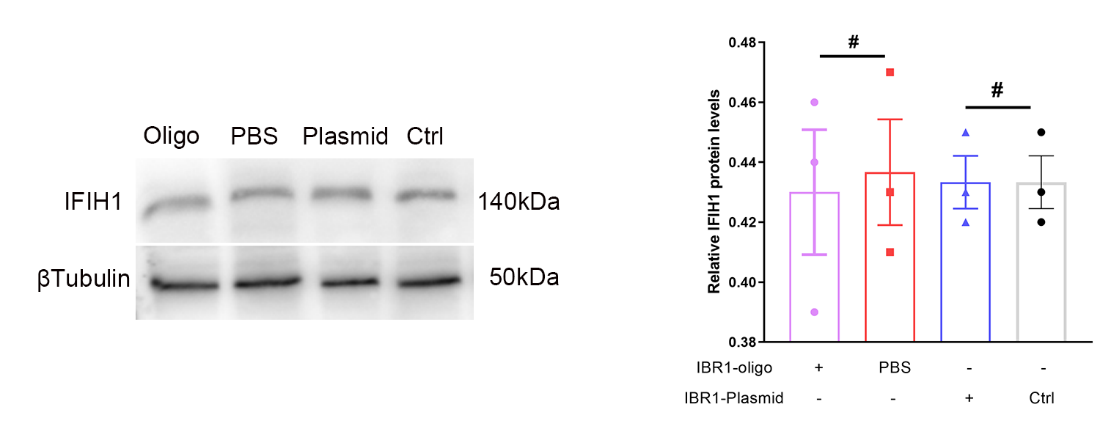


**Figure S10. IBR1 has no effect on the expression of IFIH1**


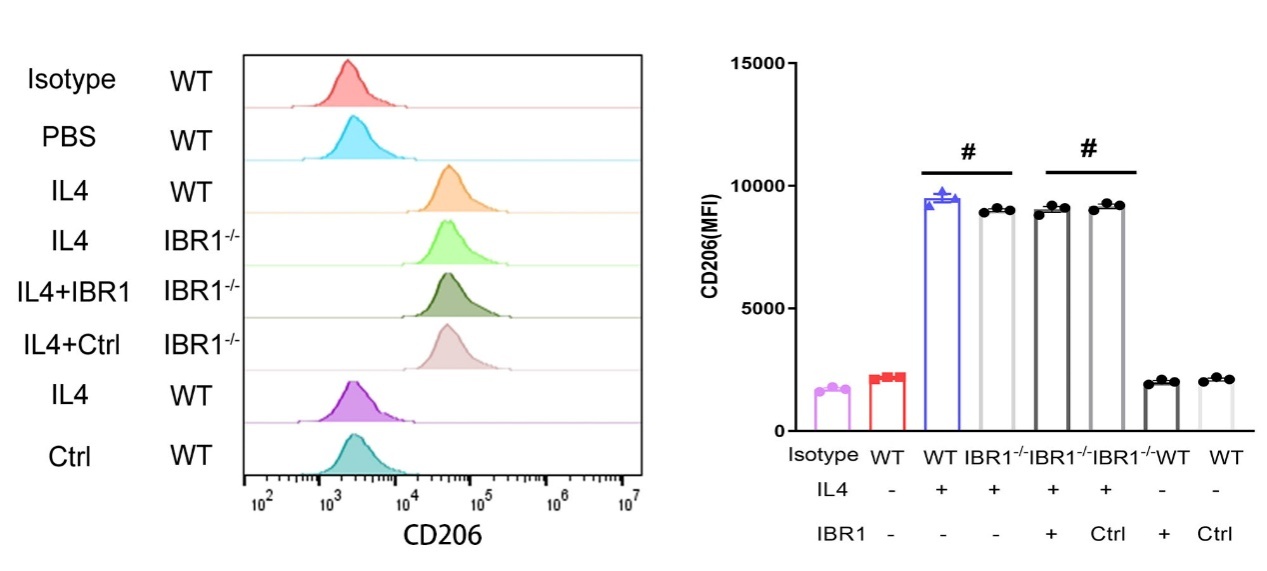


**Figure 11. IBR1 has no effect macrophage M2 polarization**

Flow cytometric analysis was conducted to measure the production of CD80 and CD86, the markers of M1 polarization, by BMDMs isolated from WT and IBR1-/-mice. The cells were stimulated with LPS (500 ng/ml) for 24 hours, with or without IBR1 overexpression.


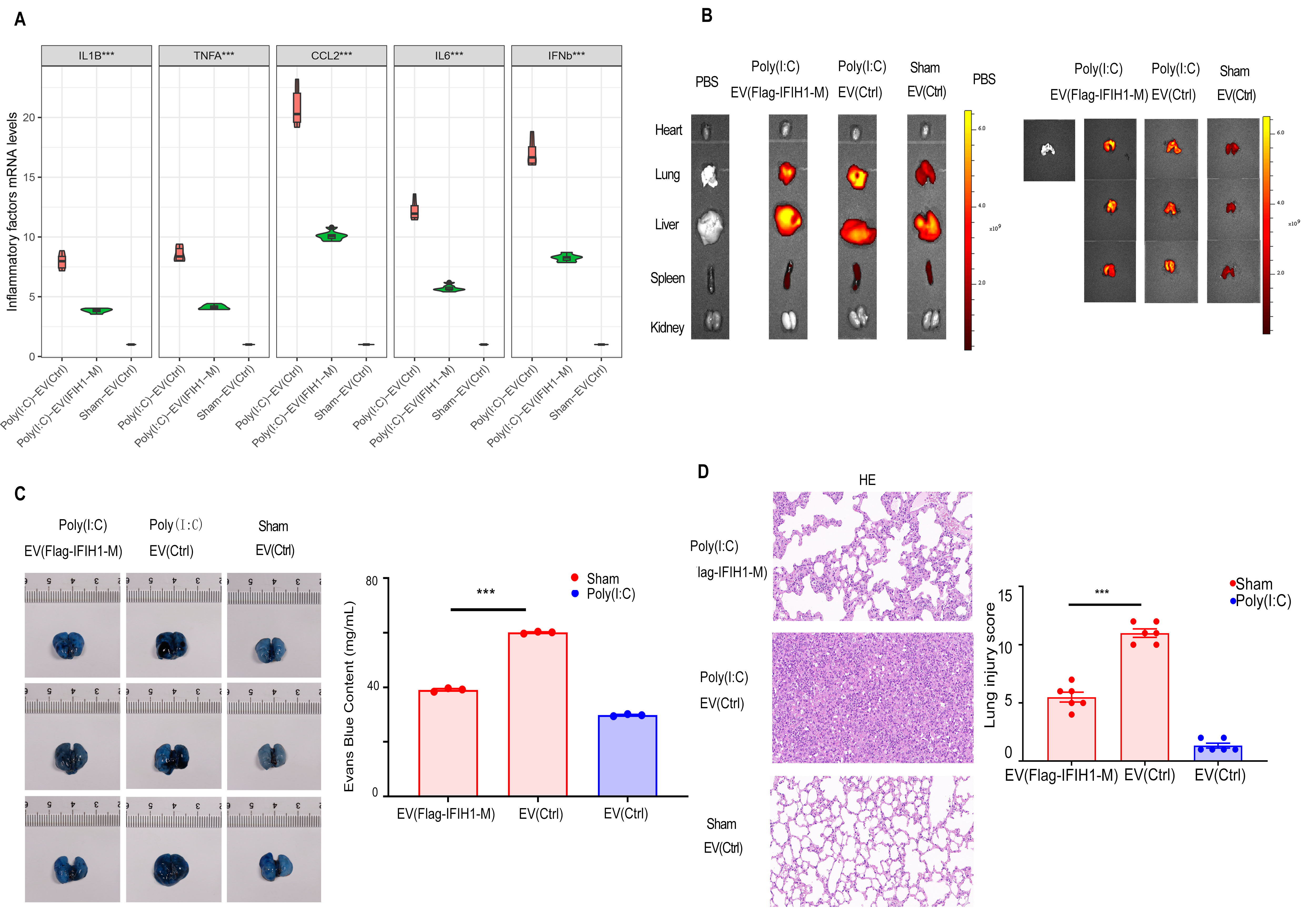


**Figure S12. Neutralization of IBR1 as a Therapeutic Approach for Poly(I:C)-ARDS**

(A) A representative fluorescence image illustrates the distribution of DiD-labeled EVs in various organs across different groups following injection for 24 hours.

(B and C) The impact of IFIH1-M-EVs treatment on pulmonary vascular permeability in Evans Blue assay.

(D) Histological evaluation using H&E staining was performed to assess pulmonary injury in subjectstreated with IFIH1-M-EVs compared to untreated subjects in an Poly(I:C)-induced ARDS model. Lung tissue from the designated experimental groups was analyzed for lung injury score, serving as histological indicators.

(E) qPCR was performed to evaluate the levels of pro-inflammatory cytokines in pulmonary tissue homogenate.

Table S7. Sequences of New Transcripts

| XLOC_030107-IBR1 |
| --- |
| TGTATTTTCTCCTTATAATCACTCCCTCCCGGGGCATGTAATTAATTTCCTGATGGATTACTCACACGGTCTTGAAGATGCAATGTCAGCTATTTAGGCTGTGCTAGGACTCAATTACAACAACATATGCATTAAGACAGGAACTGGCAAGCCGGGACTACAGCAACTACAGGGCTGGTGCTTCCCAACCGCTGGGCTATAATCTATGAAACTGAGCCGGGATCCAGCCAATCAGTTAGCTAGCTCCTCATAACAGGTCTAACTGGCTCTGGACAGCTAAGGGCTGCGCTGGAACGTTCCAGCGCAGCCCTTA |
| XLOC_007489-IBR2 |
| GTGAAAATGAGGTAGGGAAATTGTTATTTATCACAGAAATCCCAGAATTAATACTAGAAGACCCCAGTGAAGCCAAAGAGAACCTCATCCTACAAGAAACATCTGTGGTAGAGTCACTGGCTACGGATGGGAGCCCAGGACTGAAATCAGTGCTGTCTACAGGCCGAAATCCAAGCAACAGCTGTGACTCAGGAGAGAAACCAGTGGTCACCTTTAAAGAGAACATTAAGCCACGAGAAGTGAACCAAGGAAGAAGCTTTCCTCCCAAAGAGGTAAAATCCCAGACAGAACTAAGAAAGACTCCAGTGTCTGAAGCCAGGAAAACGCCTGTCACTCAAACTCCAAGTCAAACGAGTAATTCTCAGTTCATCCCCATCCATCACCCTGGAGCCTTCCCTCCTCTTCCCAGCCGACCAGGTTACACCTTCCCAGCTGGTGTTTCTGTCCCAGGAACCTTTCTTCAGTCTACAGCTCACTCTCCAGCAGGAAACCAGGTGCAAGCTGGGAAACAGTCCCACATTCCTTACAGCCAGCAACGGCCCTCTGGACCAGGGCCAATGAACCAGGGACCTCAACAGTCACAGCCACCTTCCCAGCCACCCCTTACATCTTTACCAGCTCAGCCAACAGCACAGTCTACAAGCCAATTGCAGGTTCAAGCTCTAGCTCAGC |
| XLOC_007197 |
| CUGUCUGUUAAUUCGUUCUGGUCACAGAACUUUAUUGAGGGGCGGGGCUCCGACAUCUGCACAGCUACAACUGAAUCUCAUGGUAGGCCCGCUUCUCCACCAACUUCAUUUUUUAUUUGCGCUUGAACUUGGCUCGUUCUGGUAGUAGUAGUCUUCUGGAAGCUCUUGAACCUGUCUUGGAGAAUGUGACCUUCUGGUUUCGAGUGUCCUGAGUGGGCCAGACAGCUCAGAGCGAUGGACGGAUACGACCU |
| 1600014C10Rik |
| GGGACTGCTCCCTAGGCCTCTCTGGATTTTTCTGTTACTCTGCTCTCTTTCTGAGTGGCTCCCTTTGCCCTGCAAGTGGAGTGGGCTCTGTTCTGGTCTTAGGAGGTCTGAAAGCTATACCAGCCCCTGCGCGTCCCACGCAGGCCCCAGCGTTAATTAACCAGCAGTCGCTCCTGACAGCCAAGCCCCTCTATCATCTCCCAGGTCCAGGACCCTGTGGGAGAAAGGGTCCGGCAAGTCACAGACCCAGCCAGCGCAAAGGGCGATCGAGGAGAACCTTCCAGGAACCGTCCCAGATCCTAAACGTGAGGTTGGGCACTGAGTGGGGTGGCCAGGCGGAGAGGAAGTGGGGGCAGCGGGTCCTCGCGACACAGAGTCCCCTCCCAGATGCTCCCCATTTCCGCAGGCACAGAGCTTCCTAGCCTGGGTCTTGGTGTCAGTCAATCAGACGCTGGGGCGCGGAAGGGACGCCTCGCCCCGCCATCAAGATCTTTCCCCAGGCCTGTCCCATCTTGAGGACCCAGACTCATGGGGACCCAGAGCTCGGCGACCCGGAAGGCCGGCGGAGCCGGAAGGTGGGAGGGCGGAGCCAGGGTGGCGGTGGCCTGTGAACGCCTCTCTGGAGCTGGCAGAGCAGAGCCGGCCTCAGTGCCTTGGCGGTCCCAGACTCCAGCTCGGTAAAGGTGCGCGCGGGGAAGCCGGGGTCTGCGAAGCCGGTCGGGGACCCTACGGTGTCAGGCCCAGTGCTAGAGCTGTTCATCTCAATGCAAAGGACCTGAGACACTGAAGGCCTAAGCTGCAGCGCCGAGGTTGGAGTAGTCAGCAGCTCCGTGCGCTTCCAGCCGCAACCTGGATGCTTGGGAGTGCGCGTGCGCGGTGCGGTGTCTTCCCACAGTTTATGCCTTCTGCCTAGCGGACTGCTGTGCTTGGCCCAGAGAGGGAGGAAATCTGGGCTCTAGCTTAGGGATGGCCAGGAATGAATGTCAGGGGATCAAAGGGCCAGATTAATTGGAGCTGTTGTTGAAAAACGTTCTTCTATGACTAAGTAGATAAGGTGCATGCATGGCCGGGTGCAGAGGGCACGGTGCTGCTGAGTGATGGGTGTCAGTTAACTCTGCGAGCTATGTAGCGAACCTACCACTGACCACTTCTGATCTTTCAAGAGAAATTAGGAAGATCTAAGATATATGTAAGATTGTATTATTGCTGTTAGTTTGAGACTGGGTCTCACTATGTAACCTTCACTGGTCTTGATCTGGGAGTCCTACCATAGCCTCCCCCATGCTGGGGTTATGGGAGGGAGCCAGCACACCCTCGATATCTTCTTATTTTTAAATACCAACAATCAAGTAAGTAAACAACTTAGTAGGCTGGAAGAACAGGGTGATGAACTTCACAGAGGACAGAGTACAACTGGGGAATTCCAGACAGGGGAGCAAATATTGCTATTTGGCCAAAGACCTGTTTTCCACTCAGTTTCTGTTTGTTTCTCCCTGCAGCCTCCCCTTACCCACCCCCTACCCCTGACACATTTCCTCCCTCTACTCTGGGGATTTGAATCCCAGCTTCGTCTAAGATTGCTATAAAGAACATGGAGCCCCCTGTTACTCTGAGGCCTCCTCCTCTGTTCCTCACAACTTGATTGGACTCAGGTCAGGCTGCTGTCCAAAGGTTTGAGTTTTTCACCTCCTCTGTGTATGGGAGCCCTGCTGTCATCTTCCCTCCAGTCTGGGTGACTATAGGACATCAGGGCTCCCAGTTAGCTTTTGTCTTCCCTATGGCTGGCAGATGGACTTCCGGTGAACAGGACCTGTTTTGAGGACAGCATTATTGAAGTCGTTCTGCTCCCGTGGGAAGGTCCTGTCTTCTGCGCCCCCCATCTCTTCCTCGCAGACAGACAGAGGTGGCCCCTGGAAAGAGGAGAATGGGGCGTGTTACCTTTCAATTTCTTGAGCCTGAA  GAATTCAGAAAGTGAAAGCTTTCACATAGACTCCAGGTCCCAAACCTTTCTTTCACCACAAAAGCCTGTTGCTGCCATAACTTCTGGAATTACTCTTCAGGAAACCTAGGCCAGCCTTGCCTTGCCTTGGAGACTGCAGAAGCCCAGCTGGGGCGGAACCTTTCTTGCCCGGCTCTCCTGCCCCCTGACACTTGCTAAGGAGCCTGGCTTGTCTTCTGGCAAAGGCTTGTCTGGGCGTGGTAGAGATTGAGAATGGCATGTGCATGTTGGAACCTTTTCTTTTCTGCTCCATGGGCTTTGACTTAATGTTATTCAGAGCAAATCTGGAGGTAGATAATTCTTTGAGTAGGTAATCAGGGGTAAGATGGGTGAGTCCAGTACTCATTCCCAGCTCCCTCCCCCCACCCTGCCCACACTTTTACATTCTGTAGTTTCGGCTACTCACAGCCAAGTAGTAAGTGAAAAAAAAATTCAGAAATGAACAGTTCATGAGTTTGAAATGCTATGCAAGTTTTAAGTCATGGGCTGTTCTATATGGTTAGAAATTTCATTCTGAGTAGTAGTAGTAGTAGTGATGAGCTCAAGTAACCTCGTTTTATTTAACAATGGCCCCAAAGCTTAGGAGTTACAAGGTCAAGAAACCAGAGCAAAAAGGCTGGCTGGACGGATTGACCGTACTTTCAAGCATCTGCTGGAGTCTCCCTGTGTGTTCCTTATGGATGA |
| 4930599N23Rik |
| CTCGGCCGGGTTCCCAGCACCCACATAGCAGCTCACAACTGCCTGTAACCCTGGTTCCAGGAGGGATCTGACACCCTCTTCTGGACGCTTCAGACACCAGGCACGACAGGTGTGAAGGCGCCTTTAATCCCGGCACTTGGGAGGCAGAGGCAGGCAATCTCTGAGTTCTAGTCCAGTCTGGATTTGAAAGTGTACACAGTAGGATGTTTTTGAGTGAGCATGAAGCAAACCCAACACTTTGGTCACAGTGGAGTGTGGGAGGTACTAGGAAAGTGGAAAAAGGAAAGGTGAAGGCTGAAAATTGGGAGGTGAGTGCTTAGAAAATTAATTTAAAATGAATAAAATTTTATTCTAAGTATAAAATACTACAGAAAAGAGCAACTGAGGGTATAGCCCCTGGGCTCAATCCTTAGGAAGGTGTGTGCTGGGGGGGGGGGGGAGTATTACTACAAAAATGTAATAAAATGGGATTAGAGTAGACCAATTGGCAAACCCTGGAACCCAGTGAGAGATTCTGTCTCAAAATAAGGCGGGAACTGGAGAGATGGCTTAGCAGAGAAAAGTACCTGCTGTCCAAAGCCTGCCAGCCTGAGGTGGATCCCCAAAACCCAGGTAAAAGGGGATGGAGAGAGAAACACCCCACACCCCCAAAATTGTCCTCTGACCTTCACATGTGTGCTGTGGCACACACATACATATACATAAATAAACTTAAAAAAAAAAAGGCGGAAAGCTCCTGAGAAATGACATCCTACCACACACACACCACAAAAAAAAAGACAGCAGGCTGGGCCAGAAACTCAGTGGTAGACTGTATAAACATACAACACATACAACACTAGGCTCAATTTCCCAGCAGCAACCCTTCTCAAAAAAGAAAAAAAAAATGAGTATAAAGACTAGATTTTGGGGGCTCAGCAGTTAAGAGCACTGACTGCTTTTCCAGAGGTCATGAGTTGTGGAGAACTTTCGGGCCACTTGGCAGGACATTGGCCAAGGACAAGGAGATGGGCTTCAGACAGGAATCTGACATTGGGCCATTACAAGGAAGTAAACTCAGGCAGGAATCTAATTTTAGGCTAGAACAGGGAAGTAGGCTTCAGGCATGAATATGACTTTGGACCAGGCAGTGGTGGCGCACGCCTTTAATCCCAGCACTTGGGAATCCCAGCACTTGGGAGGCAGAGGCAGGCGGATTTCTGAGTTCGAGGCCAACCTGGTCTACAGAGTTGAGTTCCAGGACAGTCAGGGAGAAACCCTGTTTCCACCCCACACCGCAGATGAATCTGACTTTGGGTTAGGATGGGGAAGTAGGCTCCGATATCTTGGTCCTCCTGATCAGCCCTTAGGAATGGTGATCACGGGACTGTGTTTACTGCCTTGCTTGCTCCTTGACTATTTGTGTTTATTGTACTGCCTGACCTTACTACTTGCATGTAAATAAAATGGTATAAAATCTGATTGGGAAAAAATAAACCTGCCTCAGAACTGGCTTGGGTCATGCTACAATGTTGTCTAATTGTCTTTTTTCTTTTTAATCCTCACTCCTGCCCTAGAAAACCTGTTGACCGACTGAGCTGGCTTGGCTCAATTCTCAACAACCACATGGTGGCTCACAACGGTCTGTAATGGGATCTGCTGCCCTCTTCTGCTCTGTCTGAAGAGAGTGACAGTGTACTCATATACGTTAAATAAATAAATAAATCTTAAAAAAATTAAAACTAGATTGTTAACAAGGACAAATGAGAATGGATTAGAGGTGAGGTGGAGGGCAATTCATTTTCTTGGACTATTTTCCATGGTTTTGATATTTTGATAAATATTTTTATTTAGTTTAGCCATTTAACTGTTTACTTGTACTTTTGATATTGCTTTGCAGTAATACCTTATTATTTTCATTCTTGGATGTCAAACAAGTGCTCTTCCATAAACATTCCCCCGTTTTAGTACCATGACTGGCTGAAAAGGCAGCGTCAGTAAAATGGTGGGTCCTTCCACAAAAGCAGTGAAGAAGCTGGCAAATCCTAGAACCTTTTCAGAGCTCTGGAAGTTTACTAAAAGCATGCAGCATCTCCTCCACAGTTTAATGGAGAAAGAAGCCAACTCTCGGTGAGAGTAGCCATTTCTGTAGCATTTTCTCCTACTCAAGTTCTATCTTAACCCTGAAGCTCTTTGCTGTCTTTGAAAAATGACAACTCACATTCCTAATGTCTAAAGCAAGGGAGCAGACAGGGTTGGAGTTCTTTCAAAGGCTCATCCCAAGGGCCACTTCTAAAAAGGCAGGTCTTTATGTGGTCTCATTGGAACTAGCAAAGTCATAACAATGCCCTTACCCTCGCAAGAGGGCATAATTATTAAATATTTTAATATCATGGCCAAAGCAATGAATAGCGGTTGAGGAAAACAATAGACTAACCAAAAAGCTGAGCAGTTCAGGGCACTGAGAGTGTGAGAATACTGTAGGAACCCACACAGGCACGTATGCATGCATAGGACCACTAAGATGCTCAGGAAACAGAAGTTCTGTCTGACACTCAGGCCCTTTGCCTGCAGGAAGCAAAAGCTATGCATACTTGCAAACTGCCTGACTATGGGAGGCATTTACCAAAACATTCATAGAGCTCCTGGACAAAGACCAAGATGGATGCTGGTTACTGGTAATCTCCATCCAGTCATTAACTGAGAGGTAAGCTAATCAAGTAGTCACACAGTGCAGATGCAAATAAGCTAAACAAAATGAGTTTGGAAAAGTCACTAGACAAACACAGTGACTACTTCATCAAGCAACAACAGCCCCAGGAAAAGAAGAAAATATGACTTTGGAAGCTGCACATTATTTGAAATGTCCATCTTCAAGTAAATTGTGACATGTATAAGGAAATAAAGTGTGACCCATACAAAGGAAGTCAAAGCTAGGAATACGAACAGCTCCTAGAGCTTGGAGAGATGGCTCAGCAGTTAAGAGCACTGGCTGCTCTTCCAGAGGACTCAGGTTTGATTCCTGGCACCCACGTGGCAGCTCATAACCAACCATAACCCTTGTACTGAGGAATCCCATGTCCTCTTCTAGCTTCTGTAGG |
| 4933412E12Rik |
| TAGGAGGTTAGGGGCCCGGGGAGGGGGGGGGGGGGGGGATGGGATGGGCGGGGAGCTGGTAAACCCAGATCCTTCCACTTTCAGTTTCCAAAGACCGTCTCTCATCTTTTACAGGAACTATATCTATTGTTTTGTTTTGTTTTGTTTTGCTTATCAAACTTCCATCCCACATTCGGGCTTCCAAGTTGGAAAACAAAAAACAAAGTACAGACAGAATCAAGCCCTGAAAATCAAACTCAGGGATGGGGGGTGGGAGGGAGCAGTCATCCTCGGTATTTCCGCCAATGTTTGTGATACTCAGAGGCAGGGGCTGCCATTCTGTTGAGTACATCAAGGAGGTGGTAATAAAAAGGAAGCTATGTGTGTGGTCCCTGTGATCAGCCACCTACTGACCTCTGTGACCTTAGGCGAGTTCATCTGCAATATCGCCAATCTTCTCACTGATAATTGGAAATAAGAATGTCTACCTCATCAAGGTTTTGAGAGTTAAGTGGTTTAATATATGTAATTAGTGTACCTCACCACATTCTGTGACACAGTGAGTCATACAGAAACACACATGTGTACACACACACACACCTCTACAAAAAATTCCTACCATCCAAAGACTCAAACAAAAGGAGACAAGTTTACTATTAAGAGAAAACTTTATGGGCTCCCTGTTTTACACATTTTTTTTCTAAAGAGATGTTCACACACACACACTTTTTGTTTTGTCTTGGTTTTCGAGACAGGGTTGGCCTCGAACTCAGAAATCTGCTGCCTCTGCCTCCCAAGTGCTGAGATTAAAGGCATGCACCACCACTGTCCGGCTCACACACACATCTTTTATCATATGGAATTGGTAATTACTTAACCCATGCTCCAGTTTCTTTGTAAACCATTCCCTGATGCTCGCCTGCCTCCCAACCCTAACTCCTTACCTGTGATCCCAGAACACTGTATTTCAGTGGTTCTCAACCTTCCTAACACTGTGACCCTCTAATACAGTTCCTCATAGTGTATACTAACCCCCAACCATAAAATTATTTCCGTTGCTACTTCATAACTGTAATTTGGCTACTGTTATGAACCTTAACATAAATATCTGTGTTTTCTGATGTCTTAGGTGACCCCCTGTGAAAGGGTCATTTGATCCCAGAGGGGTTGCGACCCACAGGTTGAGAACTGCTGCTTTAACTCTTGCAGTGCATATTCCCCATGAATCATAAGTAGACTTTCCTCTCTTTTCTCTTTAGTCTGTGTCTTGAGAACAGGCACTCTGCTTGACAGAGAACTCTGTAAAGGTAGGAAGAGGAGATGGTTGACACACAGGGAGAATGCTCTATTTGGTATATGAACATTTGGAGGATTGGCTCTTCAGTTTATTCAATGTAAGACCCAAAACATCACGGGTCTACAGAAGACAGTAATGCTAATGTGTGTGAATCCCACAGTGACCCACATGTATGTTAACTACAAGATCATCTGTAGTCTTAGAGAGTGGGCTCTAGCACTGTTTGAGAATGGGGAGAGTTACTGTAGAGCCATGACTAGAAATGGCTCCCAGAGGCTCATATATTTGGAGCTTGGTCATTGGGAATTATTTATATTATAATTATTATAATTGGGTCCTTGACAGGGATTAGGAAGTGTGGCCTTGTTGGAGGAAGTGTGTCACTAGGGGTGGACTTTGAGATTTGCTTGGGTGACCTCCACCCAAGCAAGCTTCCAACTTGTGAAAAGGAATTTATAAAAAGCTTAGGGGTTGGCATAGATGCACACACCTCACATCCCAACACCCAGAGAGCAAAGGCTGGTGGATCTCTGAGTTCCAGGCCAGCCTGGTCTACACAGTGAGTTCCAGACCAGCCTGGTCTACAAAGAGCCTAAGCCAGACCCTGTGTCTCTCTCTTCCTGCTGCCTGTGGATCTGGATGTAGATCTCCCAGCCCATTCTCTAGCATCATGTCTGCCTGCATGCCACCAGACTCCCCTCCATGGCAATGATGGACTACATTACTGAACCTGTAGACCGGCCCCAGTTAAATGCTTTCCTTCGTAAGAGTTGCCGTGGACACAGTGTCTCTTCACAGCAATAGAACATTGACTAAGGCAGAAGTTGGTACCAGGAGTGGGGTATTGTTATGACAAGCCGGGTTTCTTGGTGCAATATGGACTTTGGAACTTGGATTGGGGAAAGCAATTGAACATTTTAAGCAGGACTTAATGGGCCATCCTATTAGGTACACGGAAGACAGAACAATGTAGATTATGACCCAGATCAAAAGGTTTCAGAGGAGAAGAATATTAGTAAGTGGCCCAGAGATCATACTTGTGATATTTTGGTGAAGAGTGTGGCTGTTTTCTGCCTTTGTCCAATATACAAATAAATAAATACATACATACATACATACATACATACATACATACATAAATCTGAGGCTAAATTGAAGAGTTTTAGATTAATGGTATTGGCAGAGGAGATTTCAAAACAGCCAAGTATTGATTATGTCAATTATCATATTAGTTATTAGTGGCCAGTCTTAGGCAGATCTATAATGAAAAGAAAATGAGCTATGCAGTTTGAGGGAAAAAAGAGCACCAGGAAGTGCAATGGAGCTAAAGTCCAGGGCTCAAGAAAACAAAAGGTTTAAAGAAAAGCCTGCTGCCAAATGGACTAAAGGGAGTAGTGCCCTCAGAGCAAGACCTCCACCCAAGCAAGCTTCCAACTTGTGAAAAGGAATTTAAAAAAAGCTTAGGGGTTGGCATAGATGCACACACCTCACATCCCAGCACCCAGAGAACAAAGGCTGGTGGATCTCTGAGTTCCAGGCCAGCCTGGTCTACACAGCGAGTTCCAGACCAGCCTGGTCTACAAAGCAAGGTCCAGGACAGCCAGGCTGAAGCAGTGAAGGAAAATGTATTTGACCAAGGGGGCCAGGTTCCAGCCCCAGCAAGCACACAAACTTGGCAGCTTCGGCCACATGGTTCTGGCTTTAGAGGAAACGATACCAGAAACTCTGGAAGGATACAAGCAGCTCTGGAAATTCCTGTACAAAAACATCTGGGAACTGGAACATCCACTGCTTTGCAGCGTTCTTCATTAGACCGGCTATAGCCAGGTCTGCATTTGAGGCCACTTCTATGGTTCAAGAAAAAGGAAATAAGAGATTTAGAGGGTTGAAGGCTTCACAAAGACCTTAGACTCTAAATTGTATAGTTTCTTCTACCTGCCCCCCCCAGCCATTCATTAAACAAATATGTATTTCTCTATACAATAGAGAGGACTTCCTAGTTTTACAGAGTACAGTCTCCTTACCCCCTCAAAATACTTAAAGTAATTCTCTAGTTTTGCTTAATATAGCTTTGAAGGGATTGCTGTTACTGGTCACCAGAAAAATATTCTTTTCATGTTTTTTTTAATCTTGCTTAACATTTAATTAATTGTTTATATGGATAGAGGGTGATGCGTGCCCTGACACGAGTATGGAGGTCAGAGGACAGTTTTCAGGAGTTGGTTTTCTTCCACCATGTAGGCCCCAAGGATTGAACTTAAGTCATCAGATTTGGTGGCAAGCACCTTTACCTACTAAGCCATCTTGCTGGCCCAGAAAATACTCTTAGACAGTGTATTTCTTATGATCCAAGCAAAGCAGTATACGCTCGTAACAGCAGCACTCTCAAGACTAAGGCTAGAGGATTGTGAGTTCAAGGCCAGCCTGATCTACAGAGAGGTTTTGTTTTGTTGGTTTTCTTGAAGATGTACATTCCAAATAGCGAGTGCACAGGCTAGACCTTCAGTGTTCAGTAACGAGACCGACCTGATCCTGATCAGTAGTCAGGGGTTGCCGGCAGTGGAAGAACAAAGAGATGCTCTGTACCCACTGAAGACGAAGGCTATGGGCTGACAAGCCTAGCATCGGTGGTCAGCCATTTTGCCATTAGTGCTATGAAGAGAAGGCCTTTTCCTCAGGTAAGAACAGGATGTTATGGCTGTCAGTGTTCTTCACACCCATGCTCCTAACAGTTTCCAATTAGGGCAAAACCAAACCAGTTTGAGTTTGTTTTAATGCAGTCAGAATCCTAGACTCTGGGATCTTGTTAACATGTTTCAAGGTGAAGGGCTTTGGGGAAACTACAGAACTCTCACGGGCCTAACTTATCCAACACACAGGGACCCAAGAGAATGTGCTTTCTTGCTGTCCTGATGAATTAGCAGAATATTCTGATCTTGGAGCAACAGTCGGGGAAGGGAGCGGAGGGGCGGGTTTCAGGATATGAAGAACGCTAAGCTTAAACAAGGCATGTGGATTGAGCCCAGAGCTTAGTGGAATTACGGATTCTGAAGACCTATCCTATTTCCGTAGAAACAGAGTCCAAATTTCAGAAGAGACATTTTCCGGTGAACTTGAATCCTACTTTAAGTTTCTGCAGCTGCTTTTGGTACAAAAACTTCAACATGTGTTTGAATCACCAAGCACCTCTCTGATGCGTGCATAAGGAATTTGATTTTACGAATCTGAAAAAGGGACTATTCTACCAGAAGTTAAATTGGAATTTGAGAACTGGAATGTGAATGACAAAATTGTCCTAAATGTTAGCCTGCAGGGATATCTTTCATAACAACACTGAAATTCAGGCAGAAAGCCATCATCCTGTCAGCAACAGCAACAACAAGTCTACATTCAGCAACCCACCACCCGAACACAGTTCAAGTCACGCCACAAACATAACACATTAGCCAACAAGTTTCTGATTTATGATAATTTTTTCAGGACCAGAAGTGCTCTGCGCTCTGCTATTAACCCCATGAAAAGAGTTCTTTCCAGATAAGCGGATACTTGCGGCTGCGTGGAGTGGATCCCAGTCCCAGTTTGCTACCAGCAATCCCAACCCAAGACACAGGTGAGCCACAGGATTGTAGAGTCTGGGCTTGAGGGGAATAAACA |
